# A state-of-the-art methodology for high-throughput in silico vaccine discovery against protozoan parasites and exemplified with discovered candidates for *Toxoplasma gondii*

**DOI:** 10.1038/s41598-023-34863-9

**Published:** 2023-05-22

**Authors:** Stephen J. Goodswen, Paul J. Kennedy, John T. Ellis

**Affiliations:** 1 School of Life Sciences, University of Technology Sydney, 15 Broadway, Ultimo, NSW 2007 Australia; 2 School of Computer Science, Faculty of Engineering and Information Technology and the Australian Artificial Intelligence Institute, University of Technology Sydney, 15 Broadway, Ultimo, NSW 2007 Australia

**Keywords:** Parasitic infection, Computational models

## Abstract

Vaccine discovery against eukaryotic parasites is not trivial as highlighted by the limited number of known vaccines compared to the number of protozoal diseases that need one. Only three of 17 priority diseases have commercial vaccines. Live and attenuated vaccines have proved to be more effective than subunit vaccines but adversely pose more unacceptable risks. One promising approach for subunit vaccines is in silico vaccine discovery, which predicts protein vaccine candidates given thousands of target organism protein sequences. This approach, nonetheless, is an overarching concept with no standardised guidebook on implementation. No known subunit vaccines against protozoan parasites exist as a result of this approach, and consequently none to emulate. The study goal was to combine current in silico discovery knowledge specific to protozoan parasites and develop a workflow representing a state-of-the-art approach. This approach reflectively integrates a parasite’s biology, a host's immune system defences, and importantly, bioinformatics programs needed to predict vaccine candidates. To demonstrate the workflow effectiveness, every *Toxoplasma gondii* protein was ranked in its capacity to provide long-term protective immunity. Although testing in animal models is required to validate these predictions, most of the top ranked candidates are supported by publications reinforcing our confidence in the approach.

## Introduction

An aspirational goal, first conceived in 2000, was the ability to predict protein or epitope-based vaccine candidates entirely on a computer (in silico) without the need for cultivating the target pathogen in a laboratory^1^. This goal was inspired by the technological success of protein sequencing at the time, plus the draft sequencing of the human genome^2^. A new revolutionary approach named reverse vaccinology (RV) was devised to realize the goal^3^. In the 22 years following the emergence of RV, the research community witnessed an exponential increase in pathogen sequence data that is freely available in public databases; an exponential increase in computer processing power, digital storage capacity, and digital communication and networking; an increase in freely available software to analyse the sequences; and the maturing of bioinformatics and machine learning (ML) to aid in this analysis. RV as a result of these technological advances has evolved and merged with other in silico related approaches such as subtractive proteomics, computational vaccinology, and immunoinformatics. The term ‘in silico vaccine discovery’ is used henceforth to encapsulate all approaches. Fundamentally it is a methodology for the identification of antigenic components, sourced from the disease-causing pathogen, to create a subunit vaccine. A question arising from our current historical perspective is whether the goal of predicting vaccine candidates entirely in silico has been fully achieved. This question is especially pertinent to protozoan parasite subunit vaccines, which is the research focus of this article.

One rigorous measure of success is whether an in silico vaccine candidate has made it all the way through to licencing. Supplementary Table S1 lists all vaccines licensed for use in the United States, irrespective of the discovery approach (source: The United States Food and Drug Administration). No vaccine has yet been licensed in the USA for human protozoal diseases. Seven vaccines, however, are available for protozoal diseases of animals (see Table 1). Five of the seven are subunit vaccines of which four are for leishmaniasis (or leishmaniosis) in dogs. All seven vaccines were discovered following traditional laboratory approaches. This underscores that there are currently no known subunit vaccines against protozoans as a result of an in silico vaccine discovery approach.

**Table 1:** Current licensed vaccines for protozoal diseases of animals.

Vaccine	Target host	Target protozoan	Type	Disease	M	R
Leishmune	Dog	*Leishmania chagasi* and *Leishmania infantum*	Protein and carbohydrate chains	Leishmaniasis	L	^4^
CaniLeish^a^	Dog	*Leishmania infantum*	Excreted secreted proteins	Leishmaniasis	L	^5^
Leish-tec	Dog	*Leishmania infantum*	Saponin and recombinant protein A2	Leishmaniasis	L	^6^
Letifend	Dog	*Leishmania infantum*	Recombinant protein Q	Leishmaniasis	L	^7^
Coxabic	Chicken	*Eimeria spp.*	Transmission-blocking vaccine composed of affinity-purified antigens	Coccidiosis	L	^8^
Toxovax^b^	Sheep	*Toxoplasma gondii*	Live-attenuated vaccine (tachyzoites)	Toxoplasmosis	L	^9^
Giardiavax	Cat/Dog	*Giardia lamblia*	Inactivated trophozoites	Giardiasis	L	^10^

*Leishmania chagasi* and *Leishmania infantum* are from the phylum Euglenozoa, *Toxoplasma gondii* from the phylum Apicomplexa, and *Giardia lamblia* from the phylum Metamonada.

Key to columns: *M.* method used to discover vaccine (*L* = laboratory i.e. not an in silico discovery method), *R.* published reference.

^a^CaniLeish contains an ‘adjuvant’ (a highly purified fraction of *Quillaja saponaria*) to enhance the immune response.

^b^Available in Europe and New Zealand from MSD Animal Health: https://www.msd-animal-health.co.nz/products/toxovax/; and administered to avoid abortion in sheep. Toxovax has been reported to decrease the abortion rate but does not eradicate the parasite. It is not considered an appropriate vaccine for humans because of its live attenuated formulation has the potential to revert to a pathogenic form.

Supplementary Table S2 lists 17 priority protozoal diseases in need of a vaccine as compiled from publications authored by the World Health Organisation (WHO), Food and Agriculture Organization of the United Nations (FAO), and the Bill and Melinda Gates Foundation. Only three of the 17 diseases have commercial vaccines (as per Table 1). This further highlights that there are no vaccines for most protozoal diseases, irrespective of discovery method. A more appropriate opening question might be why there are so few protozoan vaccines despite decades of research? Table 2 lists some possible answers as proposed in several reviews^11–14^. Palatnik-de-Sousa and Nico present a comparative history on the delay in licensing of protozoal vaccines^13^; and McAllister reviews protozoal vaccines and their designs, including descriptions of three subunit vaccines yet to be licensed^12^. Live and attenuated vaccines, so far, have proved to be substantially more effective than novel vaccine designs such as subunits^14^. However, the unacceptable risks posed by live and attenuated vaccines means that there remains a critical need to research alternative designs^13^. The current strategy to improve efficacy of subunit vaccines and increase their protection term is to use appropriate adjuvants and have regular booster vaccinations, respectively.

**Table 2:** Challenges facing vaccine discovery against eukaryotic parasites.

Challenge	Impact
Complex multiple life cycle stages yet to be fully understood and/or studied in detail	
Expression of protein antigens can be different at each life-cycle stage and under altered environmental conditions, such as interactions with a host during infection	Choice of candidate can be specific to a life cycle stage
Antigens (in terms of protein sequences and/or 3D structure) can vary over time e.g. ancient and ongoing interactions between protozoans and the immune system have influenced their coevolution	Efficacy of candidate can change over time
Parasites are always mutating. Mutations can change parasites in ways that allow better resistance to immune defences and opportunities for multiple mechanisms of immune evasion e.g. mutations introduce variability in vaccine targets	Efficacy of candidate can change over time
In the endeavour to greatly improve safety, advances in vaccine candidates such as subunit components tend to be less immunogenic or efficacious than traditional live, whole pathogen vaccines	Requires potent adjuvants and appropriate delivery vectors
In a laboratory discovery approach, the expression of proteins is different in vitro than those proteins expressed during infection in vivo. Furthermore, abundantly expressed proteins are more easily identified in the laboratory	Potential vaccine candidates are missed
Unknown contributing factors that may be detrimental to vaccine discovery e.g. contributions from definitive and intermediate hosts, and transmission vectors (all complex biological systems)	
Limited knowledge of precise interactions between parasites and host immune system, but more specifically, the interactions between antigens and immune cells	Difficult to assess the contribution of candidate to overall efficacy
Types of immune responses needed for protection is not completely understood for many protozoans	
Different strains of parasites have different levels of virulence	Protective immunity may be better in some strains than others
No standardized testing protocol	A quantitative comparison of claimed protection levels between studies is difficult
Coinfection with other pathogens can influence the host response to vaccination	
For any protozoal disease, methods must be developed to cultivate the pathogen	Some pathogens are too difficult and/or dangerous to cultivate in the laboratory
Many protozoan diseases are exacerbated by poverty and challenging environmental conditions in developing tropical and subtropical countries	
A protozoan disease needs to attract the attention of vaccine manufacturers e.g. some diseases maybe considered commercially less profitable than others	Influences research and development for vaccines
Geopolitics can play a role in which diseases obtain the necessary funding	Influences research and development for vaccines
Regulatory limitations and safety concerns can impede vaccine licensing, especially for human recipients	
The number of protozoan diseases in need of a vaccine is not static due to changing factors such as the rapid population growth in areas with weak health systems, climate change, antimicrobial resistance, and the changing nature of pathogen transmission between human and animal populations	
For targeted human diseases, inability to test vaccine candidates on humans during vaccine discovery	Reliance on natural animal disease models
Species barriers to clinical translation e.g. antigens identified in mice may not protect humans or food-producing animals	Efficacy tested on animal model may not translate to target host
Limited research funding, although more funding is available for human diseases	

Apicomplexan parasites are acknowledged as the most prevalent and successful intracellular pathogens^15^ i.e. this phylum of protozoan parasites actively invade host cells and cause disease. A notable apicomplexan infecting more humans than any other protozoan on the planet is *Toxoplasma gondii*, which causes the disease toxoplasmosis. This organism mainly gained attention because of its association with birth defects in humans^16^ but is now considered an underestimated threat^17,18^ commanding even more attention. *Toxoplasma gondii* was chosen in this study as the target to evaluate state-of-the-art in silico vaccine discovery. This choice was guided by several facts: it is an important model system for the phylum Apicomplexa^19^, well-studied with 13,137 scientific publications with ‘*Toxoplasma gondii*’ in the title (source: Web of Science, January 2023), and crucially for in silico vaccine discovery, has protein sequences from multiple *T. gondii* strains readily available. In-depth information on *T. gondii* can be found in reviews elsewhere^20–23^.

Although there is only one known vaccine against toxoplasmosis (see Table 1), there are numerous publications reporting *T. gondii* vaccine candidates with efficacy evidence from various testing strategies in animal models. Many of these candidates are listed and/or discussed in recent reviews^11,14,24–27^. Invasion of host cells is an essential life cycle event for the survival of *T. gondii*. Therefore, proteins playing a role in this event have received the most research attention and consequently, the most represented candidates in publications. In brief, an apicomplexan pathogen invades a host cell first by recognising host-cell surface receptors via antigens on its cell membrane, and then secreting proteins from specialized secretory organelles (rhoptries, micronemes and dense granules)^16,28^. Table 3 lists the most represented protein candidate types in publications, namely surface antigen (SAG and SRS), rhoptry (ROP), dense granule (GRA), and microneme (MIC) proteins. The most common similarities between these candidates are their association with *T. gondii* virulence^21^ and their tendency to be naturally exposed to the immune system. Researchers for the popularly published candidates are assumed to have focused specifically on proteins with these latter characteristics because they were traditionally expected to make the best candidates. These candidates (as per Table 3) are referred to henceforth as ‘classical vaccine candidates’. It remains unclear as to whether they specifically possess a distinctive characteristic or whether any exposed protein could be considered for candidacy. Furthermore, multiple factors can impact the efficacy of a vaccine formulation e.g. differences in method of antigen preparation, mouse models, ages of mice at the time-point of infection, vaccine delivery routes, adjuvants, vaccination and infection doses, parasite culture systems, challenge strains, and immunogenicity assessments. This means that a quantitative comparison of claimed protection levels following vaccination is difficult and consequently, assessing the actual contribution made by each published protein candidate is unfeasible. A further point adding to this contribution uncertainty is that none of the vaccine formulations in the respective publications achieved the desired long-lasting protection against toxoplasmosis, including prevention and elimination of tissue cysts and/or fully blocking vertical transmission. These candidates, nonetheless, could conceivably achieve better or worse results given different factors. Furthermore, an encouraging driver for continuing efforts to obtain a vaccine is that a natural infection from *T. gondii* elicits protective immunity against reinfection in most animals, including humans^14^.

**Table 3:** Classical *Toxoplasma gondii* vaccine targets.

Protein type	Role	Life cycle stage^a^
Micronemes (MICs)	Recognition, attachment, and penetration of parasite to host cells	All
Rhoptries (ROPs)	Discharged into cytosol to interact with host cellular organelles and are important for the biogenesis of the parasitophorous vacuole during the parasite invasion	All
Rhoptry neck proteins (RONs)	Secreted into the host cell membrane to help microneme proteins	All
Rhomboids (ROMs)	Trigger microneme activity	All
Apical membrane antigens 1 (AMA1)	Cooperates with RONs during moving junction and host cell invasion	Tachyzoite
Dense granules (GRAs)	Modify the parasitophorous vacuole and are thought to contribute to nutrient gain for functioning in intracellular survival and replication	All
Superfamily of surface antigens (SRS)	Precise role unclear, but possibly act as surface protein adhesins to facilitate entry into host cells.	All
Surface antigen glycoprotein (SAG) family	Smallest member of the SRS superfamily	Tachyzoites, bradyzoites
Oocyst wall proteins (OWP)	Provides strong protection from harsh environmental conditions	Oocysts
Matrix antigen 1 (MAG1)	Secreted into the host cell and suppresses (modulates) the host immune reaction (a GRA protein found in the parasitophorous vacuolar matrix in tachyzoites; and cyst wall and matrix in bradyzoites)	Tachyzoites, bradyzoites
Heat shock protein (HSP)	Regulates key signal transduction events and plays an important role in growth, development, and virulence	Tachyzoites, bradyzoites

^a^There are three pathogenic (infectious) forms of *Toxoplasma gondii* related to its three asexual life cycle stages: tachyzoites (rapidly multiplying form), bradyzoites (formed in tissue cysts), and sporozoites (formed in oocysts). Merozoites only occur in the sexual stages of reproduction in felines such as cats. ‘all’ = a protein from this protein type can be expressed in any of one of the three infectious stages but not necessarily in all stages e.g. GRA1 is expressed in all three zoites stages but GRA2 only expressed in tachyzoites and bradyzoites.

Collectively, the candidate publications indicate that the immune correlates of protection and the exact type and intensity of response are still unclear. Therefore, an ongoing challenge is ascertaining the obligatory immune response to a vaccine formulation that would induce the desired protection. Protective immunity to *T. gondii* is complex and has been extensively reviewed^11,14,24,29^. Succinctly, the innate immune system, and both humoral immunity and cell-mediated immunity (CMI) respond at various infectious stages in an attempt to control a *T. gondii* natural infection. Our primary focus here was determining the initial immune system component that is the cascade catalyst to subsequent immune and antimicrobial responses e.g. the vaccine antigens need to induce *this* initial component in order to replicate the same protective host immune response during *T. gondii* invasion and infection. The expectation is that CMI components (i.e. T-cells) will be the main players because *T. gondii* is an obligate intracellular protozoan, and T helper (Th) lymphocytes will play a central role in the principal effector mechanism. Moreover, humoral immunity (B-cells and circulating antibodies) can only play a role in controlling extracellular parasites^25^. Th cells are essentially cytokine factories directing other immune system players by secreting subsets of chemical messengers (cytokines). There are two main subsets, named Th1 and Th2. Pattern‐recognition receptors and cytokine receptors on dendritic cells (a type of antigen presenting cell) detect invading pathogens and display a mixture of co‐stimulatory molecules on its surface which depend on the type of pathogen encountered. Whether a naïve Th differentiates into Th1 or Th2 depends on which cytokines are produced by dendritic cells (DCs) in response to the type and location of the pathogen. From a subunit vaccine design perspective, whether to trigger a Th1 or Th2 response against *T. gondii* is a crucial decision. There is still some published debate, but the consensus proposes Th1^14,22,29^. The main cytokine types produced by activated Th1 cells are interferon gamma (IFN-γ), interleukin-2 (IL-2), and tumor necrosis factor (TNF). TNF helps activate macrophages and natural killer (NK) cells, IFN-γ maintains macrophage activation and influences B cells to produce antibodies; and IL-2 (a growth factor) stimulates the proliferation of cytotoxic T-cells (CTLs), NK cells, and Th1 cells themselves.

**Figure 1:**
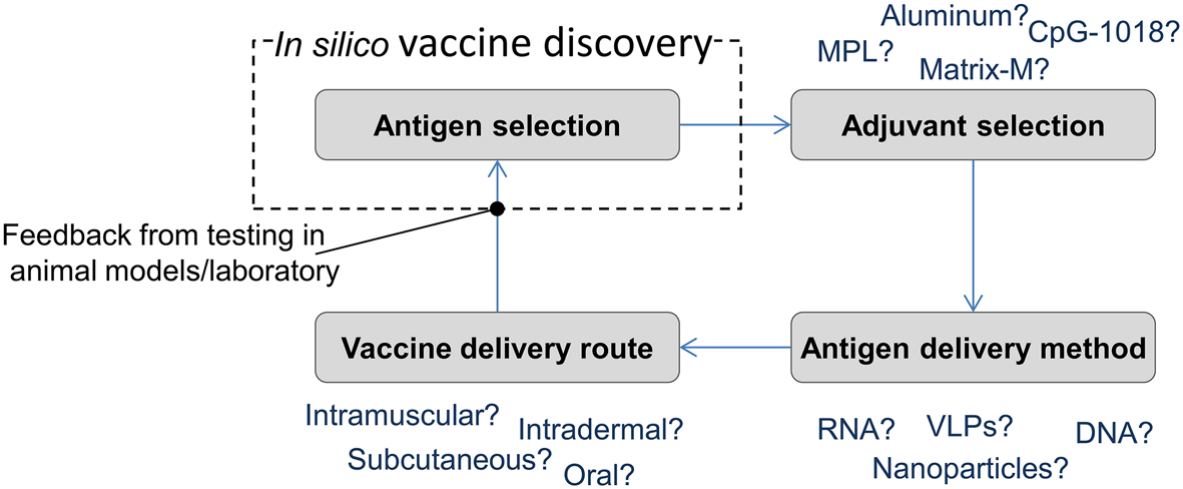
Four critical interdependent vaccine design decisions. Key:Adjuvants—Aluminum denotes one or more of the following: amorphous aluminum hydroxyphosphate sulfate (AAHS), aluminum hydroxide, aluminum phosphate, potassium aluminum sulfate (Alum); Matrix-M indicates saponins derived from the soapbark tree (*Quillaja saponaria* Molina); MPL abbreviates for Monophosphoryl lipid A and is the first non-alum vaccine adjuvant to achieve widespread acceptance; CpG-1018 abbreviates for Cytosine phosphoguanine (CpG), which is a synthetic form of DNA mimicking bacterial and viral genetic material. Vaccine delivery routes (administration routes)—Intramuscular = into muscle layer; Subcutaneous = into the subcutaneous, or fatty layer beneath the skin; Intradermal = into the outer layers of skin, between the epidermis and the dermis; Oral—taken in either tablet or liquid formulation. Antigen delivery method VLPs = virus-like particles.

There are essentially four critical interdependent vaccine design decisions (see Fig. 1). The first crucial step in vaccine design is the precise selection of antigens, and this is the principal focus of the current study. The second decision is the adjuvant type. Various adjuvants with different purposes have been used for decades in vaccine formulations, but the molecular mechanisms by which these adjuvants work remain unclear^30^. The choice of adjuvants is limited as only six have been included in licensed vaccines^31^, and the choice also depends on the desired adjuvant purpose. Our interest here is how a vaccine formulation can mimic a *T. gondii* invasion such that the immune system recognises it as both foreign and dangerous. Adjuvants that are toll-like receptor (TLR) agonists seem to be a potential solution in providing the requisite danger signal i.e. these types of adjuvants contain pathogen-associated molecular patterns (PAMPs), which can activate TLRs (a type of pattern recognition receptor) on DCs. MPL (a modified version of the bacterial surface protein LPS) and CpG-1018 are TLR agonist-based adjuvants currently used in licensed vaccines^30^. A strong humoral response was detected at 3 weeks post immunization with recombinant *T. gondii* GRA2 protein combined with a MPL adjuvant^32^. The third decision is the type of antigen display i.e. the delivery vehicle. The decision is influenced by the required immune response. A crucial event for a CMI response is the antigen uptake by DCs. Otherwise, antigens will not be presented to Th cells. The decision here is which carrier or delivery vehicle for multiple antigens (and possible adjuvants) should be utilised to enhance DC phagocytosis. Possible choices are nanoparticles (NPs), liposomes, DNA, and RNA delivery vehicles. These vehicles are reviewed elsewhere^33–36^. No studies on *T. gondii* RNA vaccines were found, but several are discussed in a recent review^37^ on *T. gondii* DNA vaccines. Except, this review also reports that none of the studies demonstrated complete protection. Vaccine components for a humoral response ideally need to be presented directly to the immune system in their native 3D structures to mimic a natural infection, but as a compromise, the repetitive presentation of multivalent antigens on the surface of a carrier could increase both their affinity and the binding to B-cell receptors to invoke the desired humoral response. For example, virus-like particles (VLPs) decorated with full-length antigens^38^. Results from a recent study showed that a VLPs vaccine expressing *T. gondii* rhoptry ROP13 elicited significantly high levels of *T. gondii*-specific antibody responses^39^.The fourth decision is the appropriate vaccine delivery route (i.e. routes of administration) to ensure optimal immune response and minimal side effects in the vaccine recipient. Examples of administration routes are intramuscular, subcutaneous, intradermal, and oral (reviewed elsewhere^40,41^).

Vaccine discovery against eukaryotic parasites is not trivial as highlighted by the limited number of known vaccines in comparison to the number of protozoal diseases that need one. There are various challenges to vaccine discovery as stressed in Table 2. Solutions to several of these challenges are most likely beyond the researcher’s control (e.g. limited funding, geopolitics, and commercial decisions), but some challenges can be addressed by in silico vaccine discovery. The goal of this study is to demonstrate the capacity of a high-throughput, in silico vaccine discovery methodology to identify novel vaccine candidates for further investigation. The ‘high-throughput’ denotes that every single available protein of a target protozoan, irrespective of its current characterisation and naming, is evaluated for its candidacy potential. The ‘novelty’ of the vaccine candidates is that they aim to address known challenges in providing widespread efficacy in multiple strains and all infection life cycle stages (e.g. tachyzoites, bradyzoites, and sporozoites,), and maintaining this efficacy by using less genetically variable candidates. *Toxoplasma gondii* is used in the demonstration. Every *T. gondii* protein is assigned a score representing its merit as a potential vaccine component. Researchers can adapt our presented methodology for their protozoan target and/or select the desired percentage of high-scoring *T. gondii* proteins for further investigation in accordance with their laboratory/animal model testing capability and budget.

## Results

**Figure 2:**
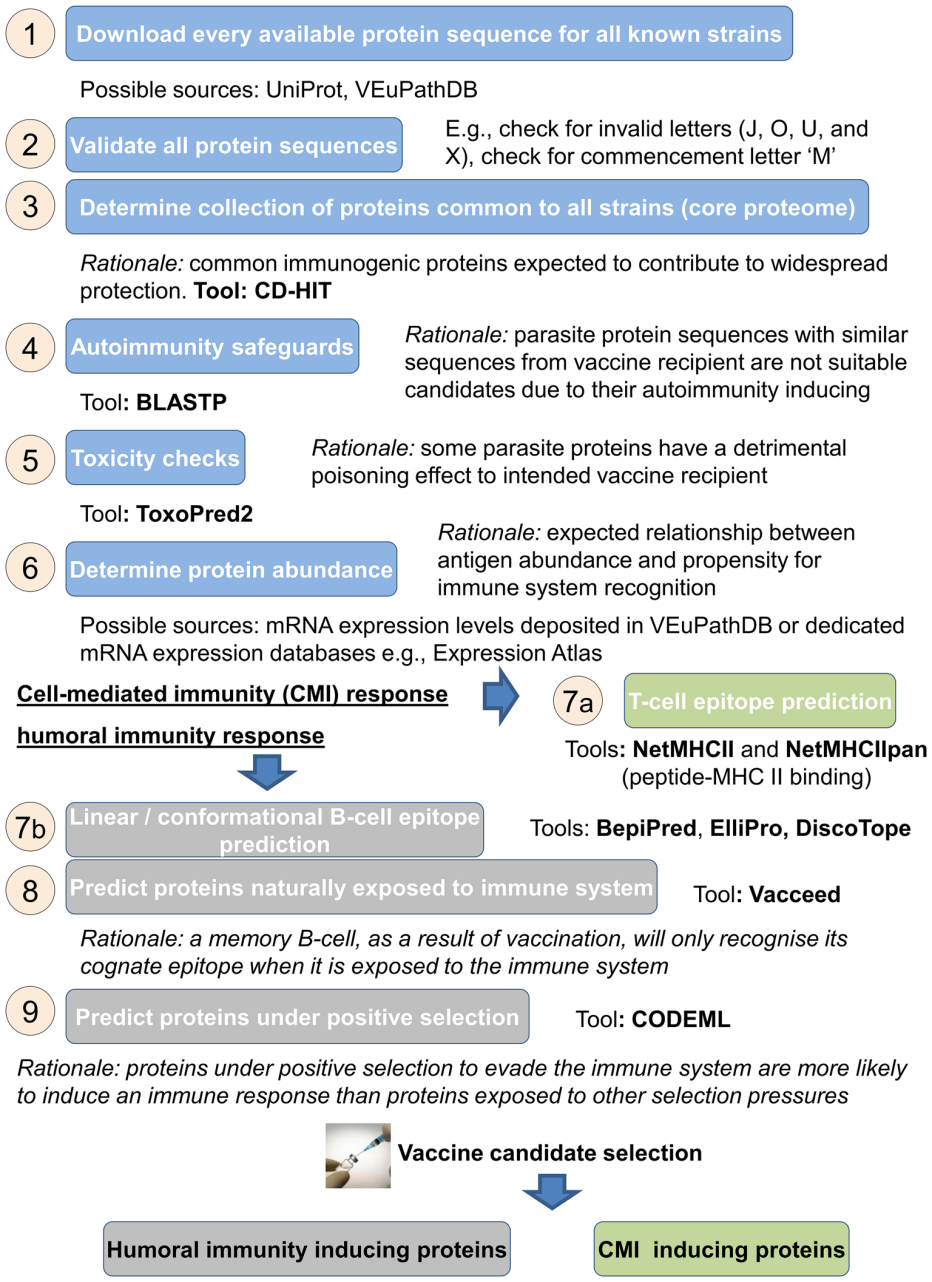
A schematic of pipeline processes for high-throughput in silico vaccine discovery. Steps 1 to 7a are for the identification of cell-mediated immunity (CMI) inducing proteins. Protein characteristics of CMI candidates: conserved among strains, contain multiple peptides that strongly bind to *promiscuous* MHC II alleles, and contain multiple *promiscuous* peptides that strongly bind to multiple MHC alleles. Steps 1 to 7b to 9 are for the identification of humoral immunity (HI) inducing proteins. Protein characteristics of HI candidates: conserved among strains, naturally exposed to the immune system, have no or minimal positive selection sites, and contain multiple B-cell epitopes.

Figure 2 shows a schematic of the high-throughput, in silico vaccine discovery pipeline applied in this study. The end goal is to identify two sets of vaccine candidates. One set designed to induce a cellular and the other to induce a humoral response. More specifically, the approach begins with every available protein sequence for the target *T. gondii* strain. It ends with two scored lists showing vaccine candidacy potential of the input sequence’s parent protein. One list is specific to CMI and the other, humoral immunity.

The CMI list is compiled from data and predictions relevant to the *T. gondii* core proteome, protein abundance, and peptides binding to major histocompatibility complex (MHC) class II molecules; whereas the humoral immunity list is relevant to the *T. gondii* core proteome, linear and conformational B-cell epitopes, protein abundance, and proteins naturally exposed to the immune system and under positive selection.

### Validation of protein sequences

Thousands of *T. gondii* strains are expected to exist but so far only 67 strains have been assigned a National Center for Biotechnology Information (NCBI) Taxonomy ID. Only 21 strains have at least one protein sequence (see Table 4) and only 15 have sequences representing the entire proteome e.g. strains PRU (1), RH (6), TgCATBr5 (1), TgCatBr64 (1), type I (2), and type II (2) have only a few proteins sequenced, as indicted in the brackets.

**Table 4:** *Toxoplasma gondii* strains that have protein sequences available.

Strain	Taxonomy	Type	Host	#. of proteins	#. reduced	Comments
ARI	1074872		Human	9958	9927	
CAST	943122		Human	9494	9455	
COUG	1074873		Cougar	9866	9835	
FOU	943167		Human	10,116	10,097	
GT1	507601	II	Goat	8445	8351	
GAB2-2007-GAL-DOM2	1130820		Chicken	9135	9111	
MAS	943118		Human	10,006	9992	
ME49	508771	I	Sheep	8315	8236	Reference strain
P89	943119		Pig	9698	9678	
PRU	1080348	II	Human	1	1	Ron10 protein
RH	383379	I	Human	6	6	
RH-88	1208370	I	Human	8316	8147	
RUB	935652		Human	10,027	10,000	
TgCATBr5	943121		Cat	1	1	Ron10 protein
TgCatBr64	1208666		Cat	1	1	Ron10 protein
TgCATBr9	943120		Cat	9833	9811	
TgCatPRC2	1130821		Cat	10,120	10,089	
type I	1209525			4	2	
type II	1209523			4	2	
VAND	933077			9252	9232	
VEG	432359	III	Human	11,148	8948	

Key to columns: Taxonomy = National Center for Biotechnology Information (NCBI) taxonomy ID; Type = types I, II, or III. In a study by Howe and Sibley^42^, the population genetic structure of *Toxoplasma gondii* was originally thought to be clonal, with most isolates belonging to one of three lineages, designated Type I (most virulent type), Type II and Type III. The three types are now thought *not* to be representative of the global *T. gondii* population; Host = organism from which the strain was isolated; #. of proteins = number of protein sequences available in the Universal Protein Resource (UniProt); #. Reduced = reduced number of protein sequences after removing identical and/or redundant proteins per strain.

All downloaded protein sequences and their annotation for the 15 strains were assessed (see ‘Sequence evaluation’ in “Materials and methods”). Supplementary Table S3 shows this assessment relevant to sequences and annotation from *T. gondii* strains ME49 and RH-88. Note that most protein sequences from all *Toxoplasma* strains are predicted, poorly annotated, and mainly uncharacterised.

### Core proteome for *Toxoplasma gondii* strains

A core proteome represents the collection of proteins common to all strains. Common immunogenic proteins are expected to contribute to widespread protection i.e. protect against all existing and potential new strains. A core proteome for all 15 strains was determined using CD-HIT (cluster database at high identity with tolerance)^43^ by creating clusters of sequences greater than 90% sequence similarity. The total number of clusters was 14,832. Supplementary Table S4 lists all the clusters. The important column is ‘No. of strains’, which indicates the number of strains contributing to the cluster. All clusters with 15 strains are considered here to represent the core proteome. Typically, the protein with the longest sequence in the cluster is used as the cluster’s representative sequence. That is, any sequence from one of the 15 strains could theoretically be the representative (see columns ‘Rep. ID’, ‘Rep. Name’, and ‘Rep. Strain’ in Supplementary Table S4, Excel sheet [Core proteome]). Note that the cluster’s representative protein name (e.g. Rep. Name) can be different to the most frequently used name in the cluster. The current study used the RH-88 sequence from each cluster as the representative, although the ME49 genome sequence is normally considered the reference. RH-88 is the most recent strain to be sequenced using the latest third-generation sequencing technology and is judged the current most accurate *T. gondii* genome sequence^44^. There are 6199 out of 14,832 proteins (41.79%) representing the clusters that are common to all 15 T*. gondii* strains (see, sheet [Proteins per No. of strains], and therefore 6199 RH-88 proteins are common to proteins in 15 strains*.*

### Autoimmunity safeguards and toxicity predictions

To avoid the likelihood of an autoimmune response and/or a detrimental poisoning effect to the intended vaccine recipient, *T. gondii* protein sequences were checked for their similarity to human sequences and their potential for toxicity were predicted. Supplementary Table S5 shows the results for all RH-88 proteins. In summary, 176 proteins have the potential to trigger an autoimmune response in humans (e.g. polyubiquitin UbC protein predicted to have the greatest potential), and 203 have toxicity potential (e.g. translation initiation factor IF-2 protein predicted the most toxic). Irrespective of these results, no proteins were discarded from further analysis. This aligns with our approach that all predicted characteristics are collectively considered to assess vaccine candidacy. Preferably, a host’s possible allergic reaction to a protein also needs to be checked. However, no standalone program for high-throughput allergenicity predictions could be found for this study.

### Determining protein abundance

There is an expected relationship between surface antigen abundance and their propensity to be recognised by the immune system. A similar expected relationship is between protein abundance in phagocytosed pathogens and their availability for antigen presentation. No clear published evidence of these relationships was found for *T. gondii,* but there is supporting evidence from bacterial and viral studies^45–48^. Protein abundance levels at different time points during the *T. gondii* life cycle stages may be an additional useful indicator for antigen selection e.g. antigens that increase in abundance during host infection are likely by association to have higher epitope presence for recognition, and hence score more favourably as vaccine candidates. Two different datasets of abundance levels were ideally required for this study: one to indicate a protein’s abundance prior to cell invasion, and the other to indicate a protein’s abundance prior to proteolytic fragmentation in a DC. These datasets were not forthcoming in any known public database. Measurements of mRNA expression levels, however, were used here as a compromise. A caveat is that due to post transcriptional processing of eukaryotic proteins, mRNA levels are not an exact determinant of protein abundance^49^ i.e. protein and mRNA abundance data correlate poorly^50^. Results from eight publications^51–58^, related to RNA sequencing (RNA-seq) and specific to *T. gondii,* were compiled to show metrics of absolute and/or changes in RNA expression levels for captured *T. gondii* proteins. Supplementary Table S5 lists this compilation. Proteins with the highest RNA expressions in tachyzoites and sporozoites are mainly the classical vaccine candidates (especially GRA1 and SRS29B). These expression levels appear to be irrespective of whether the RNA captured time point was intracellular or extracellular. The protein with the highest RNA expression in bradyzoites is BAG1. RNA expressions that changed the most when comparing samples from tachyzoites and bradyzoites were *not* the classical vaccine candidates (except some SAG-related sequences had reduced levels e.g. SRS29B). The main purpose of the compiled RNA expression was to provide a comparative indicator during antigen selection e.g. a protein associated with high or increased RNA expression at a particular life cycle stage was weighted more favourably than one with fewer expression levels, if the compared protein from the same stage had other equivalent candidacy indicators.

### Evaluating peptide-MHC class II binding predictors

One of the vaccine design requirements was to produce a Th response. To obtain such a response, T cell receptors (TCRs) on Th cells need to recognise antigen peptides presented by MHC class II molecules on professional antigen presenting cells (APCs) e.g. macrophages and DCs. The aim here was to evaluate programs that can predict peptides that have an affinity to MHC class II molecules.

Nine peptide-MHC class II binding predictors (MHCPred, RANKPEP, SYFPEITH, Vaxitop, IEDB’s MHC-II Binding predictor, MHC2Pred, NetMHCII, NetMHCIIpan, and ProPred) were assessed for suitability for the pipeline. The predictors SYFPEITHI, Vaxitop, MHCPred, ProPred, and MHC2Pred allow only one sequence at a time to be processed and provide no standalone version (RANKPEP allows only 100 sequences at a time). This means that there are only three predictors (IEDB MHC-II Binding^59^, NetMHCII^60^, and NetMHCIIpan^61^) that have the capacity for high-throughput processing.

To evaluate the three high-throughput predictors, known T-cell epitopes were downloaded from the Immune Epitope Database (IEDB). In an attempt to have the best quality test data, only epitopes associated with more than one publication over the last 10 years *and* sourced from parent proteins with ‘reviewed’ status in UniProt were used. Supplementary Table S6, sheet [Published_epitopes] lists the epitopes downloaded from IEDB. These epitopes are associated with 911 parent proteins. Sequences of the 911 proteins were used as input into the predictors. Different default thresholds are automatically applied by the predictors to prediction outputs to determine if a peptide is a strong binder (SB) or weak binder (WB) or a non-binder. Prediction outcomes are listed in Supplementary Table S6, and Supplementary Table S7 presents a summary.

There were 6201 published epitopes, which collectively represent true positives for the test parent proteins. An evaluation challenge is that we do not know how many other ‘true’ epitopes exist on the test proteins. This means that in the evaluation only ‘true positive’ (TP), ‘false negative’ (FN), and sensitivity (SN) can be appraised with any certainty e.g. a ‘false positive’ (FP) epitope could be an epitope yet to be published. However, comparing the predictors solely on SN is misleading when considering the substantial difference in false positives. For example, IEDB predicts more TPs but substantially more FPS in comparison to the other predictors. Given the number of predictions (e.g. TPs + FPs) in contrast to the number correctly predicted, it could be argued that the predictors’ precision is no better than guessing. The consolatory factor, conversely, is that we do know how many of the FPs are in fact true.

Only peptides predicted to be SBs by all three predictors were recognised here as potential epitopes. This is on the assumption that predictors in agreement are more reliable than contradictory predictions but still on the understanding that most peptide-MHC II binding predictions are likely to be FPs given their unreliable precision. Given the 6201 published epitopes, 319 true positives and 1788 false positives were predicted by all *three* predictors (see Supplementary Table S6).

### T-cell epitope prediction for *Toxoplasma gondii* RH-88

The aim was to predict the binding affinity to MHC II molecules of every possible peptide in every RH-88 protein. This is on the premise that *any* protein irrespective of its subcellular location can potentially contain peptides that bind to MHC II molecules for surveillance by Th cells. There is no guarantee, however, that the presented peptide will be recognised by a Th cell.

Given published MHC II peptides from IEDB, peptide lengths range from two to 80 amino acids (AAs) but typical lengths are 13–25 AAs. There are numerous MHC II alleles e.g. MHC-peptide predictions can be obtained for 5628 available alleles for NetMHCIIpan; and 54 alleles (25 HLA-DR, 20 HLA-DQ, and 9 HLA-DP alleles) for NetMHCII. In theory, to predict every feasible MHC binding peptide for each protein, a predictor would need to be executed for every combination of peptide length and MHC II allele e.g. for NetMHCII, the program would need to run 54 times for peptide lengths of 13 AAs, and then another 54 times for peptide lengths of 14 AAs, and so on. This means that the program would need to execute 702 times per protein for lengths 13–25 AAs. On the computer platform used in this study (see Materials and methods), the processing time for one protein was approximately 60 minutes, notwithstanding the gigabytes of output data per protein. The processing time required to process all 8147 RH-88 proteins was calculated to be potentially 330 days. Predicting every MHC binding peptide is possible but an impractical exercise.

Given more than nine million different references to MHC II alleles in IEDB, the frequency of each allele was determined. There are only 237 frequently referenced alleles. Supplementary Table S8, sheet [Published_alleles_from_IEDB] lists the frequencies e.g. DPA1*01:03 /DPB1*04:01 is the most frequent with 242110 references, whereas DQA1*01:04/DQB1*05:03 is only referenced once. Alleles that were common between the predictors and the 237 published alleles were determined (e.g. NetMHCII has 54 and NetMHCIIpan has 5628 alleles available for computation). Supplementary Table S8 lists the 33 identified common alleles that were subsequently used for the MHC-peptide predictions.

Given 442,019 published MHC II epitopes from IEDB, the frequency of peptide lengths were determined (see Supplementary Table S8, sheet [Published_peptide_lengths]). The most frequent length is 15 AAs (i.e. 14.63% of peptides have a length of 15AAs). Peptide lengths from 13 to 19 AAs represent 74% of the published epitopes.

The compromise to the previously described processing challenge was to use only peptide lengths from 13 to 19 AAs and the 33 common MHC II alleles. Therefore, the programs were executed 231 times per protein (i.e. 7 peptide lengths * 33 alleles = 231).

The section ‘T-cell epitope prediction’ in “Materials and methods” describes the concept behind a sliding fixed-sized window to calculate peptide-MHC II binding affinity, which underpins NetMHCII and NetMHCIIpan methodology. One consequence of this window methodology and a high-throughput approach is gigabytes of predictions. For example, over 35 GB of predications were generated from the ‘13 AAs window’ predictions and another 36 GB from the ‘14 AAs window’ predictions, and so on (this is for predictions covering peptide lengths from 13 to 19 AAs). Furthermore, there were 1000s of dispersed SB and WB predictions associated with the 33 MHC alleles for which many were expected to be false as per previous testing results.

The aim was to filter out possible false positives from the pipeline by establishing stringent selection criteria for a SB peptide. For example, a peptide’s binding core is typically 9AAs. Therefore, peptides predicted to be SB on *nine* or more consecutive sliding windows were counted as potential epitopes.

A further challenge was how to address the repetitive nature of AAs in sequences. Supplementary Table S9, sheet [Peptide Frequency 15 AA] lists the frequency of a particular AA peptide combination e.g. SSSSSSSSSSSSSSA is the most frequent SB combination of 15 AAs. Similarly, SSSSSSSSS is the most frequent AA core combination as shown in sheet [Core Frequency]. To address this challenge, the pipeline excludes any predicted SB peptide for which its 9 AAs core overlaps a low complexity region (LCR) on a protein sequence.

Supplementary Table S9, sheet [Counts_per_ID] shows the results when combining predictions for peptide lengths 13 to 19 AAs and all 33 MHC alleles. A normalised score was applied to each of the 8147 RH-88 proteins taking into account the total number of predicted SB peptides per MHC allele, the number of peptides binding to ‘promiscuous’ MHC alleles, and the number of ‘promiscuous’ peptides binding to multiple MHC alleles (see column ‘T-cell Rank’ in sheet [Counts_per_ID]). The higher the score the more likely the protein contains multiple promiscuous strong binding peptides that bind to promiscuous MHC alleles.

It is clear from the results that high scoring proteins tend to have long sequences. Intuitively, one would expect longer sequences to have more binding peptides than shorter ones. However, there is not a perfect correlation between high scoring and protein length e.g. examples exist of proteins scoring higher than proteins with longer length.

### Evaluating linear B-cell epitope predictors

A humoral response is proposed as the main mechanism to prevent tachyzoites from invading cells in the first place. Programs are available that predict regions of proteins that are likely to be recognized as epitopes in the context of a humoral response. Four linear B-cell epitope predictors (ABCpred, Bcepred, BepiPred, and predictors from IEDB) were assessed for suitability for the in silico approach. Known B-cell epitopes downloaded from IEDB were used as a benchmark dataset in the evaluation. Only epitopes fulfilling the following criteria were included: epitope identified in a published experiment from the last 10 years, the parent protein has a ‘reviewed’ status in UniProt, and the epitope is referenced in more than one publication.

The accuracy of the predictors ranged between 50 to 56%, although publications from the predictors’ developers report 65.93% accuracy for ABCpred server^62^ (developed in 2006), 58.7% for Bcepred^63^ (2004), and 62% for BepiPred-2.0^64^ (2017). The IEDB B-cell epitope prediction tools provide a collection of seven methods with Bepipred-2.0 as the default method. ABCpred and Bcepred only process one sequence at a time and therefore do not have the capacity for high-throughput processing required for this study’s pipeline.

BepiPred has recently been improved with version 3.0^65^ and predicts both linear and conformational B-cell epitopes from protein sequences. The authors claim in their publication that it outperforms BepiPred-2.0, which was the best out of all the predictors tested in this study e.g. BepiPred 2.0 AUC = 0.596 and BepiPred 3.0 AUC = 0.710. Sheets [BepiPred-2.0_A0A0K2GUJ4] and [BepiPred-3.0_ A0A0K2GUJ4] in Supplementary Table S10 show outputs for the different program versions for one protein (A0A0K2GUJ4, which contains a known epitope of length 32 AAs located at position 1–32). Each amino acid is scored. The programs do not indicate which amino acid is part of an epitope. The epitope decision is at the discretion of the user by choosing a threshold. The suggested threshold value by the developers is 0.5 for BepiPred-2.0 and 0.1512 for BepiPred-3.0. We cannot confirm whether the scores located outside the known epitope are false positives or epitopes yet to be identified. Sheet [BepiPred3_benchmarking] shows the results when comparing the benchmark epitopes from 186 parent proteins with the predicted BepiPred-3.0 scores e.g. 25 out of 32 residues (78.12%) representing known epitope regions in the protein A0A0K2GUJ4 were correctly predicted. On the assumption the benchmark epitopes are truly correct, 50% or more of the epitope regions were correctly predicted on 46 out of 186 proteins (24.7%). Lowering the threshold improves this result but at the expense of an unknown increase in false positives.

### Predicting *Toxoplasma gondii* RH-88 proteins naturally exposed to the immune system

An expectation is that a primed or memory B-cell, as a result of natural infection or vaccination, will only have the opportunity to recognise its cognate epitope when the epitope is completely exposed to surveillance e.g. membrane bound or secreted. The program Vacceed^66^ was used to predict a score that indicates proteins are naturally exposed to the immune system. That is, membrane-associated proteins, including those spanning or anchored to the membrane, and proteins secreted to the outside of the pathogen are in full view of a host’s immune system surveillance. Consequently, naturally exposed proteins containing B-cell epitopes are considered more likely to be antigenic than those located within the pathogen. Supplementary Table S11 shows the predicted exposure score for all 8147 T*. gondii* RH-88 proteins, and 1986 have a greater than 90% probability of being naturally exposed to the immune system.

### Linear B-cell epitope prediction for *Toxoplasma gondii* RH-88

The program BepiPred was used to predict linear B-cell epitopes for all 8147 T*. gondii* RH-88 proteins. More specifically, BepiPred scores each amino acid in a protein sequence and a higher score indicates the amino acid is more likely to be part of a B-cell epitope. The challenge was how best to collectively score each RH-88 protein given hundreds of scored amino acids. To reiterate, BepiPred does not indicate which amino acid is part of an epitope. Furthermore, there is a relationship between an optimal BepiPred threshold and protein length i.e. the optimal threshold appears to be lower for longer proteins. Finding an optimal threshold for each protein is not conducive for high-throughput processing. The strategy in this study was to first normalise the BepiPred scores for each protein to make them independent of protein length. Then, consecutive amino acids of a certain length (e.g. 12 AAs) that had normalised scores above 0.7 were identified and considered here as representative of a possible B-cell epitope. Scores of the consecutive amino acids were averaged and the average scores from each consecutive group were added to generate a single protein score.

Supplementary Table S11, sheet [Epitope_lengths] shows the frequency of epitope lengths given 123338 known B-cell epitopes downloaded from IEDB. Lengths of 12, 15, and 16 AAs are by far the most frequent. Therefore, the ‘consecutive amino acids’ strategy applied to normalised BepiPred scores was repeated for these three frequent lengths. Sheets [Length_12], [Length_15], and [Length_16] show the generated protein scores along with columns ‘AA_Count’ (the number of AAs above 0.7 threshold but not necessarily consecutive), ‘Density’ (the number of epitopes as defined by consecutive AAs of length 12, 15 or 16, respectively), and ExposureScore (a score predicted by Vacceed representing the probability that the protein is exposed to the immune system). The protein scores and density from the three applied lengths were averaged. A normalised score taking into account the average protein score and density was used as a final indicator of the likelihood a protein contains multiple linear B-cell epitopes (see sheet [Combined_results]).

### Conformational B-cell epitope prediction for *Toxoplasma gondii* RH-88

The standalone versions of DiscoTope^67^ and ElliPro^68^ were used to predict conformational B-cell epitopes. These standalone versions provided the capacity to perform high-throughput processing. Ideally, B-cell epitope predictions for all 8147 T*. gondii* RH-88 proteins were sought. However, the required program input for DiscoTope and ElliPro is a protein’s 3D structure and there are none available for RH-88 proteins. In the AlphaFold Protein Structure Database^69^ there are currently 6901 3D structures for *T. gondii* ME49 proteins that were predicted by AlphaFold v2.0^70^. Therefore, B-cell epitopes were predicted for the 6901 ME49 proteins instead. Then, RH-88 proteins that had a greater than 90% sequence similarity to the ME49 proteins were assumed to have a similar number and distribution of B-cell epitopes. The results are shown in Supplementary Table S12 sheet [Conformational predictions]. A normalised score taking into account the number of conformational B-cell epitopes predicted by ElliPro and the number of binding sites predicted by DiscoTope was used as a final indicator of the likelihood a protein contains multiple conformational B-cell epitopes.

### Predicting proteins under positive selection

The premise for this study is that proteins under positive selection (PS) to evade the immune system are more likely to induce an immune response than proteins exposed to other selection pressures. The methodology from a previous study^71^ that exploits ortholog groups was used to predict which RH-88 proteins have PS indicators. Ideally, an indicator was sought for all 8147 RH-88 proteins. However, the nature of the methodology provided an indicator for only 475 proteins. That is, only 475 ortholog groups could be created that fulfilled the obligatory criteria for a group. In the previous study^71^, the ideal ‘Goldilocks’ criteria for membership of a sequence to an ortholog group were sequence similarity thresholds greater than 70% and less than 95%. Supplementary Table S12 lists the 475 proteins that were members of ortholog groups suitable for PS detection. The important columns are ‘ExposureScore’ (the probability that the protein is exposed to the immune system as determined by Vacceed) and ‘P > 99%’ (number of positive sites > 99% posterior probability). Many of the protein names associated with the PS indicators are recognisable classical vaccine candidates e.g. ROP1, ROP18, GRA4, MIC2, toxofilin, and the SAG_related sequences. However, there are equally as many classical vaccine targets that have no PS indicators e.g. ROP5, ROP6, GRA1, GRA3, MIC1, and MIC3. This is because their sequence similarity is too close (i.e. > 99%) between the target proteins from different strains to fulfil the requirements for an ortholog group. Proteins that have high scores in ‘ExposureScore’ and ‘P > 99%’ are likely to be under continued positive selection from the immune system.

### Combining predictions to create vaccine candidate lists

For the CMI list, results pertinent to T-cell epitope prediction, RNA expression levels in the three *T. gondii* infectious stages (tachyzoites, bradyzoites, and sporozoites), sequence similarity in other strains, toxicity, and autoimmunity potential were combined and presented in Supplementary Table S13, sheet [Vaccine #1 CMI]. Given all 8147 RH-88 proteins, the aim was to rank them on candidacy potential respecting both T-cell epitope prediction scores and RNA expression levels. The ranking method is described in ‘Materials and methods’ under ‘Methodology for ranking protein characteristics’. Table 5 shows an extract of the top 10 rows taken from the CMI list. The scores have been removed leaving only rank numbers. Proteins from each infectious stage were ranked separately, and then an overall rank was determined from the three stage ranks e.g. dense granule protein GRA1 (TGRH88_081130) had the eighth highest rank in the tachyzoite stage, 24th highest in the bradyzoite stage, and the highest in the sporozoite stage when compared to all other RH-88 proteins. GRA1, however, is ranked the highest for all infectious stages. Five classical vaccine candidates are in the top 10 (3 * GRAs, 1 * ROP, and 2 * RONs) but no MIC, SAG or SRS proteins. These high ranked candidates, as with any selected candidate, need to also be considered in the context of their protection efficacy against multiple strains, and their likelihood of triggering an autoimmune response and/or a detrimental poisoning effect. Although the latter protein characteristics are excluded from the ranking process, they are in the list to help make informed candidate selection decisions. For example, Table 5 shows that chaperonin protein BiP (TGRH88_051090) and ribosomal protein RPS9 (TGRH88_062360) have an autoimmunity potential, and rhoptry protein ROP7 (TGRH88_020190) has a sequence similarity to only 14 of 15 known strains with sequences. With these known characteristics in mind, a possible judicious selection decision might be to include ROP7 but exclude the potential autoimmune triggers from further analysis. Given the top 10 names (excluding the ‘unspecified product’ proteins), poly (ADP-ribose) glycohydrolase (PARG) could be judged novel from a classical candidate perspective. PARG is a protein distributed within the parasite interior and is reported to play a regulation role in the cell cycle, including death^72^. Interestingly, PARG inhibition in *Trypanosoma* parasites has a detrimental effect on their growth^72^. Four of the top 20 names are ribosomal proteins. These protein types have been identified as targets for protection against toxoplasmosis and leishmaniasis^73,74^.

**Table 5:** Top 10 overall ranked vaccine candidates specific to cell-mediated immunity.

RH88 ID	Name	Len	Toxicity	Auto-immunity	No. of Strains	Tz rank	Bz rank	Sp rank	Overall rank
TGRH88_081130	dense granule protein GRA1	190	Non-Toxin	Safe	15	8	24	1	1
TGRH88_020190	rhoptry protein ROP7	575	Non-Toxin	Safe	14	39	2	12	2
TGRH88_046790	dense granule protein GRA3	222	Non-Toxin	Safe	15	5	59	2	3
TGRH88_050950	rhoptry neck protein RON5	1702	Non-Toxin	Safe	15	20	35	17	4
TGRH88_051090	chaperonin protein BiP	668	Non-Toxin	Potential	15	23	17	33	5
TGRH88_062360	ribosomal protein RPS9	188	Non-Toxin	Potential	15	43	15	25	6
TGRH88_077370	unspecified product	1049	Non-Toxin	Safe	15	41	34	14	7
TGRH88_043710	rhoptry neck protein RON3	1958	Non-Toxin	Safe	15	18	41	43	8
TGRH88_033050	poly(ADP-ribose) glycohydrolase	553	Non-Toxin	Safe	15	74	49	4	9
TGRH88_001930	unspecified product	519	Non-Toxin	Safe	15	60	43	44	10

Key to columns: Len = protein length; Toxicity = a ‘Toxin’ or ‘Non-Toxin’ classification indicating a protein’s potential toxicity as predicted by the program ToxinPred2; Autoimmunity = a ‘Safe’ or ‘Potential’ classification indicating the potential of the protein to trigger an autoimmune response in humans (based on sequence similarity between RH-88 *Toxplasma gondii* and humans as calculated by BLASTP); No. of Strains = number of different strains that have a protein with a sequence similarity > 90% to the RH88 ID protein sequence; Tz_rank, Bz_rank, and Sp_rank contain the vaccine candidacy rank that is specific to the infection stage of tachyzoites (Tz), bradyzoites (Bz), and sporozoites (Sp), respectively; Overall rank = a rank taking into account Tz_rank + Bz_rank + Sp_rank.

For the humoral immunity list, results pertinent to linear and conformational B-cell epitope predictions, RNA expression levels in the three infectious stages, exposure probabilities, positive selection indicators, sequence similarity in other strains, toxicity, and autoimmunity potential were combined and presented in Supplementary Table S13, sheet [Vaccine #2 humoral]. Similar to the CMI list, the aim was to rank all RH-88 proteins on their candidacy potential with respect to B-cell epitope prediction scores, RNA expression levels, and exposure probabilities. Table 6 shows an extract of the top 10 rows taken from the humoral immunity list (only rank numbers are shown). Four classical vaccine candidates are in the top 10 (2* GRAs and 2 * MICs). Two proteins, protease inhibitor PI2 (TGRH88_022410) and microneme protein MIC2 (TGRH88_037170) are predicted to be toxic. Furthermore, dense granule protein GRA6 (TGRH88_038710) and unspecified product (TGRH88_068970) are predicted to contain several amino acids sites under continued positive selection (i.e. proteins possibly prone to genetic variability). A cautious approach would be to exclude the toxic protein and the proteins under positive selection but at the conceivable expense of rejecting a strong candidate (e.g. GRA6 is the highest overall ranked protein). Given the top 10 names, putative myosin heavy chain, 3′5'-cyclic nucleotide phosphodiesterase domain-containing protein, and cyclophilin precursor are non-classical candidates. Myosin proteins in *T. gondii* are known to be essential for gliding motility and host cell invasion^75^, and a recombinant heavy chain myosin of *Brugia malayi* (a filarial nematode) was reported as a potent vaccine candidate^76^. Phosphodiesterases (PDEs) are thought to be associated with cyclic nucleotide signalling that govern apicomplexan parasite motility to actively infect host cells^77^; and PDEs from *Schistosoma mansoni* (a blood fluke) are considered potential vaccine candidates to control schistosomiasis^78^. Cyclophilin is a highly conserved and multifunctional protein, and a recombinant *T. gondii* cyclophilin induced a significant Th1 type immune response as indicated by high IFN-γ and IL-2 production^79^. Also, vaccination with *Neospora caninum* cyclophilin (combined with profilin) conferred partial protection against experimental neosporosis^80^.

**Table 6:** Top 10 overall ranked vaccine candidates specific to humoral immunity.

RH88 ID	Name	Len	Toxicity	Auto-immunity	No. of Strains	Under PS	Tz rank	Bz rank	Sp rank	Overall rank
TGRH88_038710	Dense granule protein GRA6	201	Non-Toxin	Safe	15	7	3	4	1	1
TGRH88_025740	Dense granule protein GRA5	120	Non-Toxin	Safe	15	1	1	6	4	2
TGRH88_022490	Putative myosin heavy chain	1124	Non-Toxin	Safe	15	0	4	2	10	3
TGRH88_011090	3′5'-cyclic nucleotide phosphodiesterase domain-containing protein	1065	Non-Toxin	Safe	15	0	2	8	9	4
TGRH88_022410	Protease inhibitor PI2	318	Toxin	Safe	15	0	13	5	2	5
TGRH88_068970	Unspecified product	131	Non-Toxin	Safe	15	15	9	20	3	6
TGRH88_037170	Microneme protein MIC2	769	Toxin	Safe	15	0	14	12	6	7
TGRH88_066240	Microneme protein MIC5	181	Non-Toxin	Safe	15	0	12	11	11	8
TGRH88_033800	Cyclophilin precursor	348	Non-Toxin	Safe	15	0	5	19	18	9
TGRH88_015150	Unspecified product	312	Non-Toxin	Safe	15	1	18	15	13	10

Key to columns: Len = protein length; Toxicity = a ‘Toxin’ or ‘Non-Toxin’ classification indicating a protein’s potential toxicity as predicted by the program ToxinPred2; Autoimmunity = a ‘Safe’ or ‘Potential’ classification indicating the potential of the protein to trigger an autoimmune response in humans (based on sequence similarity between RH-88 Toxplasma gondii and humans as calculated by BLASTP); No. of Strains = number of different strains that have a protein with a sequence similarity > 90% to the RH88 ID protein sequence; Under PS = number of positive selection sites as predicted by Bayes Empirical Bayes (BEB) analysis within CODEML; Tz_rank, Bz_rank, and Sp_rank contain the vaccine candidacy rank that is specific to the infection stage of tachyzoites (Tz), bradyzoites (Bz), and sporozoites (Sp), respectively; Overall rank = a rank taking into account Tz_rank + Bz_rank + Sp_rank.

We propose two different candidate selection strategies that are applicable to both the CMI and humoral immunity lists. Either select multiple candidates based on high overall ranks (see Tables 5 and 6) or select the highest ranking candidates from each of the infectious stages e.g. highest stage ranked candidates for CMI: HECT-domain (ubiquitin-transferase) domain-containing protein (TGRH88_032760) (tachyzoite stage), bradyzoite antigen BAG1 (TGRH88_070990) (bradyzoite stage), dense granule protein GRA1 (TGRH88_081130) (sporozoite stage); and highest ranked candidates for humoral immunity: dense granule protein GRA5 (TGRH88_025740) (tachyzoite stage), P-type ATPase PMA1 (TGRH88_005300) (bradyzoite stage), unspecified product (TGRH88_068970) (sporozoite stage). The expectation is that multiple high ranking candidates would be selected to contribute to a vaccine formulation, irrespective of selection strategy.

## Discussion

This study primarily focused on two aims. The first aim was to construct an in silico vaccine discovery pipeline that was specific to protozoan parasites. More distinctively, the pipeline role was to predict the most worthy vaccine candidates given thousands of protein sequences from a target pathogen. ‘Worthy’ candidates are those proteins that contribute to a vaccine formulation into stimulating a recipient’s adaptive immune system into generating memory helper T and B cells. These memory cells are easier to activate than naïve cells and can trigger a defence against future infections from the actual pathogen i.e. provide long-term protective immunity.

Creating a pipeline is not a trivial task because there is no universal agreement as to which bioinformatics programs among hundreds should be used and in what order. Essentially, an in silico vaccine discovery approach is simply an overarching concept with no standardised guidebook yet on how to implement the approach. Furthermore, there are currently no known subunit vaccines against protozoan parasites as a result of this approach, and consequently none to emulate. In this study, a pipeline workflow was presented (see Fig. 2.) which we believe represents a state-of-the-art approach to in silico vaccine discovery that is specific to eukaryotic pathogens. That is, appropriate bioinformatics programs are proposed for each step needed to predict vaccine candidates.

There are many layers of inaccuracies impacting an in silico vaccine discovery approach. For example, most protozoan protein sequences are translations from gene predictions (see Supplementary Table S3 as an example for *T. gondii*). These gene predictions are mainly ab initio, which involves predicting coding sequence (CDS) regions encoded in genome sequences. Recent reviews^81,82^ highlight that correctly identifying protein-coding genes remains error-prone. Some prediction inaccuracies might be explained by the genome sequence quality and the complexity of intron–exon gene structures. It is difficult to quantify the negative impact of sequence inaccuracies to the overall in silico approach*.* Nonetheless, protein sequences are the primary input to all subsequent pipeline programs, and it must be clearly understood, from a methodological analysis perspective, that the *T. gondii* sequences have not been verified by experimental means.

Most prediction programs generate a score associated with a predicted protein characteristic. A threshold value is typically applied to the score to classify a protein as either possessing or lacking the characteristic. This classification raises several points. First, irrespective of input sequence quality, programs have their own inherent levels of inaccuracies (as illuminated with MHCII binding predictors in Supplementary Table S7), and consequently at each pipeline stage there are an unknown number of false classifications. Second, in silico pipelines typically choose between two methodology types for selecting candidates, given protein characteristics—filtering or ranking. With a filtering methodology, a potential candidate could be erroneously discarded because of an incorrect classification due to poor predictions and/or an inappropriate threshold. We propose that ranking is the optimum methodology as presented in this study. That is, no protein was discarded from the pipeline. Instead, every score for each predicted characteristic was collectively considered, with the highest scoring proteins judged the worthiest candidates.

Ideally, ML should be utilised for selecting candidates, given the copious volumes of characteristic predictions. Unfortunately, there are currently insufficient numbers of verified protective antigens against protozoan parasites to create quality ML training data. Furthermore, ML algorithms require both positive and negative training data, and similarly, insufficient numbers exist showing a protein *will not* induce an immune response as determined by experimental testing.

To reiterate, in silico vaccine discovery is founded upon sequential layers of inaccuracies within genome sequencing, gene predictions, characteristic predictions, and candidate selection with or without ML. These inaccuracies are in addition to unsuitable choices of pipeline programs and workflow steps e.g. a program choice may be governed by its popularity or lack of choice, rather than on its merit. Nonetheless, encouraging progress continues in the incremental improvement of genome sequences, gene predictions, and within the constantly increasing choice of high-tech bioinformatics programs predicting evermore informative protein characteristics. Currently, in silico vaccine discovery remains a powerful complementary approach supporting traditional discovery methods by saving time and money in narrowing down candidate numbers for experimental testing. Given the momentum of the inevitable computational advances epitomised by artificial intelligence, a gradual paradigm shift will move the importance further from the time-consuming and expensive culture-based methods to high-throughput in silico vaccine discovery methods.

The second study aim was to implement the pipeline to predict candidates against *T. gondii*. The precise selection of antigens for a vaccine formulation is one of four critical interdependent vaccine design decisions (see Fig. 1). Any wrong decision can totally change the desired vaccine outcome. For example, the perfect antigens might be selected, but any wrong decision in the type of adjuvant and/or antigen display method and/or vaccine delivery route could negate their immunogenic potential. The emphasis here is that the in silico vaccine discovery outcomes cannot be judged in isolation because it is one of many components in an overall holistic approach to vaccine design.

Our proposal is to have two different vaccines to augment protective immunity. One specific to CMI (vaccine #1) and the other specific to humoral immunity (vaccine #2).

### Vaccine #1

Two key influences on our methodology development for vaccine #1 were first to understand the decisive event triggering protective CMI in a natural infection, and then how to replicate this event through a vaccine design. In summary, the event triggering natural protective immunity is expected to be only when live parasites are phagocytosed. In a natural infection, however, live parasites (in the form of tachyzoites) invade host cells as part of a lytic cycle for their existence and then safely reside in parasitophorous vacuoles (PVs) hidden from a host’s immune system^83,84^ (PVs are unique intracellular compartments made from host-cell membrane and modified by the parasite). Furthermore, tachyzoites can differentiate into bradyzoites that form tissue cysts in reaction to an immune response. Taken together, PVs and tissue cysts have proved to be successful survival mechanisms to evade the immune system. This evasion effectively means that tachyzoites within infected cells will not be fragmented, and accordingly, parasite peptides will not be presented by MHC class I and recognised by CTL receptors. Similarly, CTLs cannot detect bradyzoites in tissue cysts. Therefore, *T.gondii* during its lytic cycle of invasion, replication and egress, will be at its most vulnerable when not residing in PVs or tissue cysts. Put differently, *T.gondii* during the lytic cycle will frequently transition between an intracellular and extracellular position. A study^85^ reports that *T. gondii* extracellular parasites remain viable for only a limited period (six to 12 hours) after natural egress. It is unclear whether the extracellular time period before the next host cell invasion is an opportunity for the innate immune system. In such a case, macrophages are expected to be the main killing mechanism through phagocytosis of extracellular tachyzoites *before* host cell invasion^86^ or during extracellular-intracellular transition periods. Activated macrophages can in turn activate naive CTLs^87^. The expected main players in the flow of events towards protective immunity are therefore dendritic cells (displaying antigen fragments on MHC II molecules), Th1 cells (binding to their cognate antigen), IFN-γ + TNF + IL-2 (secreted from activated Th1), B-cells (antibody production stimulated by IFN-γ), CTLs + NK cells + Th1 cells (stimulated by IL-2). The crucial trigger in the vaccine design to replicate these events is antigen presentation on MHC II molecules.

Ideally, the in silico vaccine discovery approach needs to identify epitopes on antigens that will be irrefutably recognized by cognate TCRs on Th cells when presented on MHC II molecules. Disappointingly, no program could be found that directly predicts T-cell epitopes with sufficient accuracy. This may be why most so-called T-cell epitope predictors currently available use an indirect method of predicting peptides binding to MHC molecules. This means that the predicted peptides only represent *probable* candidates because there is no in silico approach to determine whether the peptide–MHC complexes will be presented by dendritic cells (DCs) and/or recognized by Th TCRS and/or produce memory Th cells.

The accuracy of peptide-MHC class II binding predictors remains doubtful (see Supplementary Table S7). The expectation is that any given antigen will likely have an unknown number of true and false epitope predictions. Our strategy was therefore not to focus on individual peptides as per a multi-epitope vaccine design but collectively on all predicted epitopes per antigen. One rationale for using protein antigens (rather than peptides) is to entrust DCs to select and present the most appropriate peptides. Also, a shortcoming of a peptide-based approach is that the selected peptides are specific to certain MHC alleles. This restricts its universal effectiveness. A protein-based approach is less restrictive because a protein typically contains many high-affinity peptides with potential to bind to a broader range of MHC alleles. As part of our strategy, each *T. gondii* protein was scored in accordance with the number of strong binding peptides that bind to *promiscuous* MHC II alleles, and the number of strong binding *promiscuous* peptides that bind to multiple MHC alleles.

When tachyzoites are proteolytically fragmented into peptides following phagocytosis, it is assumed that any peptide, irrespective of its parent protein’s normal subcellular location, has the potential to be presented to Th cells if it has an affinity to a DC’s MHC class II molecule. We speculate that the more abundant high-affinity peptides will have a greater probability of presentation. Continuing with this speculation, the optimum vaccine candidates are expected to be those proteins naturally in abundance with a high-density of high-affinity peptides.

As part of this study, we investigated predicting the fragmentation of proteins into peptides. The aim was to first predict fragmented peptides by predicting cleavage sites, and then predict binding affinity to MHC II molecules in the hope of improved accuracy. Supplementary Data S1 and Supplementary Tables for Data S1 describe this investigation in detail, but disappointingly, no reliable program (e.g. accuracy less than 66%) could be found that predicts which peptides are naturally processed by the MHC class II antigen presenting pathway. It appears no universal cleavage signal exists. However, there are possible natural groups of peptides specific to different endocytic proteases^88^, which we propose may be worthwhile investigating further.

### Vaccine #2

Although Th 1 activation is expected to also stimulate antibody production, it remains unclear as to what extent B-cells and circulating antibodies play a part in the overall Th 1 response towards protective immunity. Our strategy was therefore to generate another subset of candidates as per vaccine #2 that had the capacity to stimulate a recipient’s humoral immune response into generating memory B cells. The rationale is that antibodies triggered from a natural infection will have the capacity to neutralise extracellular tachyzoites from invading host cells. To generate memory B cells, B-cell receptors (BCRs) must first bind with epitopes on cognate antigens secreted from or on tachyzoites.

The accuracy of B-cell epitope predictions remains unreliable. Consistent with T-cell epitope predictions, an unknown number of true and false epitope predictions per antigen can be expected. Our strategy was to assign a single score to each protein collectively considering all predictions. This protein score indicated the likelihood an antigen contained multiple B-cell epitopes but was not our sole indicator for a possible humoral immune response.

An expectation is that virulent pathogen proteins, simply by the fact they present a danger to the host, will be optimum antigen candidates to induce a humoral response as a protection mechanism against the danger. It can therefore be assumed there is a correlation between virulence and protection. However, the more virulent the protein, the more likely the protein maintains antigenic variation due to co-evolutionary balancing acts with the host i.e. the main phenomenon underpinning immune system evasion is genetic variability. This might explain why some vaccines have shown excellent protection in preclinical and initial phase trials, but do not show long lasting protection in the field e.g. the original parasite targets at the time of vaccine development may have changed and are no longer recognised by memory cells. Most *T. gondii* candidates reported with evidence of efficacy are classical vaccine candidates. These candidates are expected to possess and maintain high levels of genetic variation; in effect creating a greater pool of mutations for natural selection to act upon to avoid recognition by the immune system. Viewed slightly differently, proteins under continued positive selection from the immune system are more likely suitable vaccine candidates than proteins exposed to other selection pressures. This presents a major conundrum. Possibly the most immunogenic targets are the most variable, which equates to a protective response that will not be long-lived.

We propose that proteins that are conserved among strains, naturally exposed to the immune system, have no or minimal positive selection sites, and contain multiple B-cell epitopes are worthy candidates to induce broader and longer-lived immunity. Hence, our strategy for vaccine #2 was to favour proteins that had a high protein score + high immune exposure score + *low* positive selection sites.

## Concluding remarks

Our study provided an important ranked list of *T. gondii* vaccine candidates that we believe is the first to be generated following a high-throughput in silico approach. This list is judged to be the optimum within the constraints of available data, current knowledge, and existing bioinformatics programs. The unfortunate limitation of any in silico approach is that the proposed candidates are fundamentally purely predictions. There is currently no way to know whether any of the predicted candidates will induce the desired immune response. What is encouraging from our results, which supports the presented methodology and pipeline, is that the top ranked candidates are those from the classical vaccine candidate group (see Table 3). There are 183 known classical candidates out of 8147 T*. gondii* proteins. The chance of randomly selecting a classical candidate is therefore 2.5%, which reinforces our confidence in the results. We speculate based on this confidence that the highly ranked non-classical proteins are worthy of further investigation.

We raised a question in the introduction as to why there are so few protozoan vaccines despite decades of research. The absence of vaccine contributions from in silico vaccine discovery is not necessarily because of the approach itself, but financial restrictions preventing indispensable in vivo validation of candidates, or at least testing in animal models. In silico vaccine discovery has an enormous potential in contributing to current and future vaccines for protozoal diseases. The hope is that this potential is recognised by financial stakeholders in health care and/or animal welfare because an in silico approach without the expensive experimental verification is an unfinished endeavour.

## Materials and methods

### Computer platform used for study

All experiments and data generation were performed on a high performance computing (HPC) cluster node with 64 bit kernel, 32 MB memory, and 8 cores. The pipeline was designed for a Linux operating system and has only been tested on Red Hat Enterprise Linux 7.9 but is expected to work on most Linux distributions. Python version used was 3.6.8.

### Collecting *Toxoplasma gondii* protein sequences

Proteins sequences for 21 T*. gondii* strains were downloaded from the Universal Protein Resource (UniProt) in a FASTA format. Note that sequences can also be downloaded from ToxoDB^89^. Table 4 lists the 21 strains.

### Sequence evaluation

All downloaded protein sequences were validated to ensure that they commenced with the letter ‘M’ (representing the amino acid methionine) and did not contain invalid letters, e.g. J, O, U, and X. All mRNA sequences were validated to ensure that they commenced with ATG; terminated with TGA, TAA, or TAG; contained only letters A, T, G, and C; and their sequence lengths were a multiple of three for later codon analysis. Furthermore, the mRNA sequences were checked to confirm that their codon translations matched their corresponding protein sequences.

### Autoimmunity checks and toxicity predictions

Sequence similarity between the RH-88 strain of *T. gondii* and humans were performed by BLASTP^90^. Only the pident (percentage of identical matches) and qcovs (query coverage per subject e.g. *T. gondii* sequence coverage per human sequence) from the BLASTP results were recorded for each *T. gondii* protein. A measure of each protein’s potential toxicity was predicted using ToxinPred2^91^. Syntax used for ToxinPred2: *python3 toxinpred2.py -i RH88.fasta -o RH88_toxins_all -d 2.*

### RNA expression

Fold change and/or normalized reads were collated from eight publications related to RNA sequencing (RNA-seq) specific to *T. gondii*. The normalized reads are shown by FPKM values, which is a popular metric to normalise RNA-seq reads (specifically paired-end) for sequencing depth and gene length. FPKM is the abbreviation for Fragments Per Kilobase Million. FPKM fold change is a measure describing how much RNA abundance changes between two samples and is calculated as sample #2 FPKM reads/sample #1 FPKM reads. Note (1) the publication RNA-seq experiments were performed on various *T. gondii* strains, but raw reads were mapped to the ME49 genome, (2) RH-88 proteins that had a greater than 90% sequence similarity to the ME49 proteins from the publications were assumed to have similar RNA concentrations, and (3) the life cycle stage of samples is assumed here only when it is not clear from the publication. The eight publications and their samples used in this study are now listed: (1) sample #1: acute infection (10 days post-infection—assumed here to be tachyzoites), sample #2: chronic infection (28 days post-infection—assumed here to be bradyzoites). Samples obtained from infected mice forebrains^55^; (2) sample #1: extracellular (assumed here to be tachyzoites), sample #2: intracellular (assumed here to be tachyzoites). Samples obtained from infected human foreskin fibroblasts (HFFs)^54^; (3) sample #1: day 3 tachyzoites, sample #2: day 4 tachyzoites. Samples obtained from tachyzoites that were grown for a period of six days in cell culture^56^; (4) Single-cell RNA sequencing (scRNA-seq) analysis performed—sample #1: unstressed (acute-stage tachyzoites), sample #2: stressed (chronic-stage bradyzoites). *T. gondii* parasites were grown in HFFs (i.e. the experimental host cells). Parasites were allowed to invade and replicate inside host cells for 24 h under standard conditions, and then switched to standard (for unstressed sample #1) or alkaline-stress medium (stressed sample #2)^58^; (5) sample #1: sporozoites, sample #2: tachyzoites. Samples obtained from infected rat intestinal epithelial cell^53^; (6) sample # 1: bradyzoites (28 days post-infection), sample #2: bradyzoites (90 days post-infection). Samples obtained from infected mice brains^52^; (7) sample #1 extracellular sporozoites (10 days post-sporulation), sample #2: intracellular sporozoites, and sample #3: intracellular tachyzoites^57^, and (8) tachyzoites (28 days post-infection), sample #2: bradyzoites (90 days post-infection). Sample #1 collected from invaded HFF cells in vitro, and sample #2 obtained from infected mice brains^51^.

### Clustering

CD-HIT was used to create clusters for each strain i.e. sequences that have > 90% similarity were clustered. The RH-88 sequence from each cluster was used as the representative (note: the longest sequence was chosen if more than one RH-88 protein was in the cluster).

### Evaluating peptide-MHC II binding predictors

Peptides for evaluating peptide-MHC II binding predictors were downloaded from IEDB. The selection criteria used: Epitope—Linear peptides; Epitope source—default used (any source); Host—Human; Assay—MHC Ligand (positive only); MHC Restriction—Class II; Disease: Any. Records for 470,217 epitopes were downloaded. Note that if ‘Infectious’ is used for the Disease setting, only 1997 epitopes are listed. The downloaded IEDB epitopes were further filtered to contain only peptides of length 15 AAs. Protein sequences associated with the filtered epitopes were downloaded from UniProt. These sequences were used as input to the online versions of the predictors: IEDB-MHC-II_Binding, NetMHCII, and NetMHCIIpan. The test MHC II allele was ‘HLA-DRB1*01:01’ for IEDB and ‘DRB1_0101’ for NetMHCII and NetMHCIIpan.

### T-cell epitope prediction

Ideally, this study wanted to use the standalone version of IEDB-MHC-II_Binding to enable high-throughput processing. However, when using the ‘IEDB Recommended’ method the program crashed before generating the results. The IEDB support staff acknowledged that an incompatibility exists between the executable netMHCII in the ‘directory < *install directory* > /methods/netmhcii-1.1-executable/netmhcii_11_executable/netMHCII’ and the computer platform used for the study. The ‘IEDB Recommended’ method is designed to use the best possible method for a given MHC molecule based on availability of predictors and previously observed predictive performance. There are eight possible methods: Consensus method, Combinatorial library (CombLib), NN-align-2.3 (equivalent to NetMHCII-2.3), NN-align-2.2 (equivalent to NetMHCII-2.2), SMM-align (equivalent to NetMHCII-1.1), Sturniolo, NetMHCIIpan-3.1, and NetMHCIIpan-3.2. The ‘IEDB Recommended’ uses the Consensus approach, which considers a combination of any three of the four methods (NN-align, SMM-align, CombLib and Sturniolo) if available for the MHC II allele, otherwise NetMHCIIpan is used. The SMM-align method when using ‘IEDB Recommended’ is causing the ‘Segmentation fault’ error.

The pipeline in this study used only the standalone versions of NetMHCII 2.3 and NetMHCIIpan 4.0. In effect, it is the equivalent to using ‘IEDB Recommended’ except for CombLib and Sturniolo. These latter two methods were published in 2008 and 1999, respectively, whereas NetMHCII 2.3 and NetMHCIIpan 4.0 were released in 2018^60^ and 2020^61^, respectively. The main difference is that NetMHCII only predicts peptide binding affinities to MHC molecules for which it has been trained e.g. currently 54 MHC II alleles, whereas NetMCHIIpan can predict peptide binding affinities to any MHC molecule of known sequence by uploading a full-length MHC protein sequence in FASTA format^60^.

NetMHCII and NetMCHIIpan slide a fixed-sized window (e.g. 15 AAs in length) one AA at a time from the N- to C-Terminal of each protein. At each window position, the binding affinity to a particular MHC II molecule is calculated e.g. there would be 199 15 AA peptides for a protein of length 213 AAs. A threshold proposed by the authors of NetMHCII and NetMHCIIpan is automatically applied to the affinity scores to indicate which peptides are SB, WB or non-binders. A typical result is that a protein can contain many WBs and several SBs dispersed along its entire length. However, the WB or SB peptides are not necessarily consecutive e.g. a particular 15AA peptide (window) might be predicted as an SB but move the window only by one AA and its non-binder.

An in-house Linux script was used to implement parallel processing of the two predictors (netMHCII and NetMHCIIpan) with seven different peptide lengths (13 to 19 AAs) and 33 common MHC II alleles e.g. 8147 T*. gondii* RH-88 proteins were processed with 26 cores allowing for about 325 proteins to be processed simultaneously. Total processing time was less than nine days.

### Low complexity region (LCR)

The program called SEG^92^ was used to predict the low complexity regions (LCR) on a protein sequence. All 8147 T*.gondii* RH88 protein sequences were processed through SEG. An in-house Python script extracted the locations of the LCRs from the SEG output e.g. TGRH88_04545022-34, 75-89. An in-house Python script excluded any predicted SB peptide for which its 9AA core was located ‘entirely’ within an LCR e.g. between 22 and 34 AAs or between 75 and 89 AAs. Note that it seems to be still a contentious research area as to what constitutes an LCR.

### Evaluating B-cell epitope predictors

Epitopes for evaluating linear B-cell epitope predictors were downloaded from IEDB. The selection criteria used: Epitope—Linear peptides; Epitope source—default used (any source; Host—Human; Assay—B-cell (positive only; Disease: Any. The IEDB output listed: 173,944 Epitopes, 7522 Antigens, 231,667 Assays, and 2741References. Parent protein IDs were obtained from the downloaded Assays and an in-house Python script determined the following: number of antigens (171,875), number of missing IDs (59,792), number of duplicates (164,453); and number of unique IDs (7,422). Epitopes were also obtained from the downloaded Assays: number of epitopes (171,875), number of epitopes with no parent protein ID (59,792), number of unique epitopes (123,338), number of different peptide lengths (68), and number of species associated with epitopes (566). Note that some epitopes are duplicated with the same PubMedID. The number of epitopes selected for inclusion in the evaluation test dataset was 1060 based on the selection criteria: parent protein ID = ‘reviewed’ status in UniProt *and* publication year > = 2012 *and* number of references in publications > 1.

Linear B-cell epitope predictors assessed: ABCpred server, Bcepred, BepiPred-2.0, and BepiPred-3.0. The IEDB B-cell epitope prediction tools provide a collection of different methods devised in 1978 (Chou and Fasman Beta-Turn Prediction), 1985 (Emini Surface Accessibility Prediction and Karplus and Schulz Flexibility Prediction), 1986 (Parker Hydrophilicity Prediction), 1990 (Kolaskar and Tongaonkar Antigenicity), 2006 (Bepipred Linear Epitope Prediction), and 2017 (Bepipred Linear Epitope Prediction 2.0).

### Predicting naturally exposed proteins using Vacceed

The ensemble of ML algorithms used by Vacceed generate probabilities that the ‘yes’ and ‘no’ classifications of exposed or non-exposed, respectively, are correct, but only ‘yes’ probabilities are displayed in the output. The ‘average ML score’ for each protein is the average probabilities of all ‘yes’ classifications.

### Linear B-cell epitope prediction with BepiPred

A standalone version of BepiPred (v3.0) was used to predict B-cell epitopes. Command line syntax: python bepipred3_CLI.py -i ./example_antigens/antigens.fasta -o ./example_output/ -pred vt_pred -t 0.17.

### Conformational B-cell epitope prediction

All available 3D structures for *T. gondii* ME49 (6901 in total) were obtained from the AlphaFold Protein Structure Database using gsutil (syntax: gsutil -m cp gs://public-datasets-deepmind-alphafold-v4/proteomes/proteome-tax_id-508771-*_v4.tar.). Three files were included in the download for each protein: model_v4.cif, confidence_v4.json, and predicted_aligned_error_v4.json. The ‘cif’ contains the atomic coordinates for the predicted protein structure in a PDBx/mmCIF format. A python script was used to convert the mmCIF files to a protein database (PDB) format. Standalone versions of ElliPro (v3.0) and DiscoTope (v1.1a) were used for the conformational B-cell epitope predictions, which require PDB files as input. Note that the latest web server version of DiscoTope is 3.0 (https://services.healthtech.dtu.dk/), but only ‘1.1a’ is available as a standalone version.

### Predicting proteins under positive selection

The methodology from a previous study^71^ was replicated but with up-to-date sequences and including RH-88 sequences. The key to the methodology is ortholog groups. The ideal criteria for an ortholog group suitable for PS detection were group members with sequence similarity thresholds greater than 70% and less than 95%; aligned sequences having greater than 70% query coverage (i.e. the percent that a BLASTP query sequence aligns to a target sequence); only one protein sequence per species (or strain) in the group, and the group has 5 or more members. Two different sequence similarity thresholds were used in turn to increase the number of ortholog group for PS detection (e.g. > 70% and < 95%, and then > 70% and < 99%). One caveat from increasing the upper similarity threshold from 95 to 99% is that it will introduce more closely related sequences for inclusion in the ortholog group at the possible expense of reducing the accuracy of the PS detection procedure.

### Methodology for ranking protein characteristics

Predicted protein characteristics for each RH-88 protein were compiled i.e. each protein had a set of protein characteristics. The methodology aim was to create one single score to collectively represent all characteristics for the purpose of ranking candidates.

The compiled characteristics are raw scores and/or counts specific to a program output, and importantly, they are mostly not comparable. For example, RNA expression levels are normalised RNA-seq reads such as ‘19,570.84’; and the peptide-MHC II results consist of a total count (the total number of predicted SB peptides when considering all 33 MHC alleles and peptide lengths 13 to 19 AAs), density ratio (total count / protein length), HD count (a count of the number of predicted SB sites beyond the 9 amino acid core threshold), and SB counts (the number of SB peptides per allele).

The first methodology step was to normalise each characteristic using the formula: normalised value = (value − minimum value)/range of values. Normalisation was performed such that a single value (0–1) was created to collectively represent a group of related characteristics e.g. normalised values for density ratio, HD count, and SB counts were added and further normalised to create a single value representing the likelihood a protein contains multiple SB peptides (named here ‘T-cell Rank’). Similarly, a single rank was created for linear B-cell predictions (Linear_B), conformational B-cell predictions (Conf_B), RNA expression levels in tachyzoites (Tz_RNA), RNA expression levels in bradyzoites (Bz_RNA), RNA expression levels in sporozoites (Sp_RNA), and exposure probabilities to the immune system (Exposed). Supplementary Table S10, sheets [Vaccine #1 CMI] and [Vaccine #2 humoral] shows the characteristic ranks.

The second methodology step was to obtain the product of the rank values associated with each vaccine type e.g. for CMI: Tz_RNA * T_cell Rank (the resultant normalised score was named Tz_score), Bz_RNA * T-cell Rank’ (resultant name: Bz_score), Sp_RNA * T-cell Rank’ (Sp_score); and for humoral: *RNA* * Exposed * linear_B * Conf_B (where *RNA* is either Tz_RNA, Bz_RNA, or Sp_RNA; and the resultant normalised score were named Tz_score, Bz_score, and Sp_score, respectively). The reason for multiplication rather than addition was to achieve equal importance from the contributing characteristics e.g. for CMI candidates the requirement was to have a protein that contains multiple SB peptides (a high T-cell rank) *and* is expressed in high levels (a high RNA rank). Using addition can potentially create a high comparative score even when one characteristic is low or zero. Despite using multiplication, however, a high ranked characteristic can still undesirably raise the rank of a poor scoring characteristic of the same protein. To address this problem, characteristics that had scores below average were weighted by 0.5. The effect of halving the value of below average characteristics ensured a more balanced contribution from each characteristic. The final ranks—Tz_rank, Bz_rank, and Sp_rank—are ascending sequential numbers based on the descending order of the normalised scores Tz_score, Bz_score, and Sp_score, respectively. The overall rank was determined by the ascending sum of Tz_rank + Bz_rank + Sp_rank i.e. the smallest sum is ranked first.

## Supplementary Information

Supplementary Table S1.

Supplementary Table S2.

Supplementary Table S3.

Supplementary Table S4.

Supplementary Table S5.

Supplementary Table S6.

Supplementary Table S7.

Supplementary Table S8.

Supplementary Table S9.

Supplementary Table S10.

Supplementary Table S11.

Supplementary Table S12.

Supplementary Table S13.

Supplementary Information.

Supplementary Information.

## Data Availability

All required datasets for the current study were downloaded from the Universal Protein Resource (UniProt) (see section ‘Collecting Toxoplasma gondii protein sequences’ under “Materials and Methods”). All analysis results are presented in Supplementary Information files.

## Code availability

The inhouse Python and Linux scripts used to demonstrate the workflow in this article are available via GitHub: https://github.com/goodswen/in_silico_vaccine_discovery.
