# Supplementary material for: A state-of-the-art methodology for high-throughput in silico vaccine discovery against protozoan parasites and exemplified with discovered candidates for Toxoplasma gondii
Source: Sci Rep. 2023 May 22;13:8243. doi: 10.1038/s41598-023-34863-9 (PMC10201501; doi:10.1038/s41598-023-34863-9)
Supplement: Supplementary file 2 — Supplementary Table S2. [file 41598_2023_34863_MOESM2_ESM.pdf]

Supplementary Table S2: Important protozoan parasites and their associated infectious disease

Parasite (*Genus*)[a]	Phylum	Disease[b]	Priority[c]
Babesia	Apicomplexa	babesiosis	
Cryptosporidium	Apicomplexa	cryptosporidiosis	F,W
Cyclospora	Apicomplexa	cyclosporiasis	F
Eimeria	Apicomplexa	coccidiosis	
Neospora	Apicomplexa	neosporosis	
Plasmodium	Apicomplexa	malaria	
Sarcocystis	Apicomplexa	sarcocystosis	F
Theileria	Apicomplexa	theileriosis	
Toxoplasma	Apicomplexa	toxoplasmosis	F,W
Trypanosoma	Euglenozoa	trypanosomiasis, dourine, surra	F,G,W
Balantidium	Ciliophora	balantidiasis	F
Ichthyophthirius	Ciliophora	white spot	
Entamoeba	Evosea	amebiasis	F,W
Leishmania	Euglenozoa	leishmaniasis	G,W
Dientamoeba	Metamonada	dientamoebaisis	
Giardia	Metamonada	giardiasis	F,W
Histomonas	Metamonada	histomoniasis	
Trichomonas	Metamonada	trichomoniasis	

[a]Parasite = the Genus of an organism that lives on or in a host organism and typically at the detriment of the host (Genus is a taxonomic name defining a group of related living organisms made up of one or more species).

[b]Disease = the name given to an abnormal condition detrimentally affecting the structure or function of all or part of a host organism due to parasite infection.

[d]Priority = F,G,W denotes priority diseases in need of a vaccine as determined by: (F) Food and Agriculture Organization of the United Nations (FAO) – Microbiological Risk Assessment series (https://www.fao.org/food-safety/scientific-advice/microbiological-risks-and-jemra/en/ and https://www.who.int/publications/i/item/9789240024892); (G) Bill and Melinda Gates Foundation – Neglected Tropical Diseases (https://www.gatesfoundation.org/our-work/programs/global-health/neglected-tropical-diseases) and Uniting to Combat Neglected Tropical Diseases (https://unitingtocombatntds.org/ntds/); and (W) World Health Organisation (WHO) – Ending the neglect to attain the Sustainable Development Goals: A road map for neglected tropical diseases 2021–2030 (https://www.who.int/publications/i/item/9789240010352). URLs last viewed December 2022.
