# Supplementary material for: A state-of-the-art methodology for high-throughput in silico vaccine discovery against protozoan parasites and exemplified with discovered candidates for Toxoplasma gondii
Source: Sci Rep. 2023 May 22;13:8243. doi: 10.1038/s41598-023-34863-9 (PMC10201501; doi:10.1038/s41598-023-34863-9)
Supplement: Supplementary file 3 — Supplementary Table S3. [file 41598_2023_34863_MOESM3_ESM.pdf]

Supplementary Table S3: Evaluation of protein sequences and annotation from *Toxoplasma gondii* strains ME49 and RH-88

Category	ME49[a]	RH-88[a]
Protein level[b]	19	25
Transcript level[b]	2	148
Homology[b]	1856	1777
Predicted[b]	6438	6366
Annotation 5	1	1
Annotation 4	5	6
Annotation 3	47	37
Annotation 2	1147	936
Annotation 1	7115	7336
Reviewed	5	4
Invalid start	34	0
Invalid letters	4	0
Uncharacterized protein	3786	4285

[a]values in the ME49 and RH-88 represent the number of proteins from the *Toxoplasma gondii* strains ME49 and RH-88 that are associated with the category description.

[b]the Universal Protein Resource (UniProt) annotates each recorded protein sequence with a 'protein existence' annotation, which indicates the type of evidence that supports the existence of the protein (see https://www.uniprot.org/help/protein_existence). Protein level = Experimental evidence at protein level; Transcript level = Experimental evidence at transcript level; Homology = Protein inferred from homology; and Predicted = Protein predicted. Note that the 'protein existence' evidence does not give information on the accuracy or correctness of the sequence.

Annotation 5-1 = A UniProt annotation score that provides a heuristic measure of the annotation content of a UniProtKB entry or proteome (see https://www.uniprot.org/help/annotation_score). Annotation 5 denotes the best-annotated entries, whereas Annotation 1 denotes an entry with basic annotation in comparison. However, the scores are not a measure of the accuracy of the annotation.

Reviewed = UniProtKB protein source and indicates proteins are from UniProtKB/Swiss-Prot, which is a high quality manually annotated and non-redundant protein sequence database bringing together experimental results, computed features and scientific conclusions; whereas, unreviewed proteins are from UniProtKB/TrEMBL, which contains protein sequences associated with computationally generated annotation and large-scale functional characterization

Invalid start = sequence commences with a letter other than 'M'; Invalid letters = sequence contains invalid letters such as J, O, U, and X; Uncharacterized protein = a protein with no formal annotation e.g., the protein name is typically assigned 'unspecified product', 'hypothetical protein', or 'Uncharacterized protein'. These proteins are predicted from nucleic acid sequences only and have protein sequences with unknown function.
