# Supplementary material for: A state-of-the-art methodology for high-throughput in silico vaccine discovery against protozoan parasites and exemplified with discovered candidates for Toxoplasma gondii
Source: Sci Rep. 2023 May 22;13:8243. doi: 10.1038/s41598-023-34863-9 (PMC10201501; doi:10.1038/s41598-023-34863-9)
Supplement: Supplementary file 7 — Supplementary Table S7. [file 41598_2023_34863_MOESM7_ESM.pdf]

Supplementary Table S7: Prediction outcomes from the evaluation of peptide-MHC II binding predictors

**Predictor**	**TP**	**FN**	**SN** (%)	**FP**
NetMHCII SB and WB	1304	4897	21.0	38178
NetMHCII SB	464	5737	7.5	7944
NetMHCIIpan SB and WB	1773	4428	28.6	25893
NetMHCIIpan SB	789	5412	12.7	5137
IEDB-MHCII SB and WB	4188	2013	67.5	227439
IEDB-MHCII SB	1352	4849	21.8	39607

Key: SB = strong binder and WB = weak binder in reference to the peptide-MHC II binding affinity; IEDB = Immune Epitope Database; TP = true positive; FN = false negative; SN = sensitivity (or True Positive Rate) = how often the classifier correctly predicts a positive condition when the condition is positive = TP / TP + FN; FP = false positive.
