# Supplementary material for: A state-of-the-art methodology for high-throughput in silico vaccine discovery against protozoan parasites and exemplified with discovered candidates for Toxoplasma gondii
Source: Sci Rep. 2023 May 22;13:8243. doi: 10.1038/s41598-023-34863-9 (PMC10201501; doi:10.1038/s41598-023-34863-9)
Supplement: Supplementary file 14 — Supplementary Information. [file 41598_2023_34863_MOESM14_ESM.pdf]

Supplementary Data S1

Introduction

This document is a supplement to the article 'A state-of-the-art methodology for high-throughput *in silico* vaccine discovery against protozoan parasites and exemplified with discovered candidates for *Toxoplasma gondii'.* We describe here our investigation of predicting the fragmentation of proteins into peptides. The results were disappointing and therefore not included in the main article. However, we still believe there is merit in the approach and maybe of interest to other researchers.

Contents

Introduction...1
Background...1
Testing existing programs that predict proteasomal cleavage sites...2
Developing a program to predict cleavage sites...3
Proposed methodology #1...3
Proposed methodology #2...4
Results...5
Appendix A: Text analysis...7
Appendix B – MHCII-NP_results...10
Appendix C: Predicting cleavage sites...20

Background

First assumption is that once the pathogen 'invades' a nucleated cell and resides in the parasitophorous vacuole, it is beyond the reach of the immune system. That it, the parasite peptides 'will not' be presented on MHC I molecules. Second assumption is that given the opportunity, professional antigen presenting cells (APCs) (e.g., macrophages, dendritic cells) will engulf and digest the pathogen, and present peptides on MHC II molecules for the benefit of helper T cells. That is, the engulfed pathogen proteins are proteolytically fragmented into peptides.

A proposed strategy to improve T-cell epitope predictions is to first predict the fragmented peptides (by predicting the cleavage sites), and t*hen* predict the binding affinity to MHC II molecules using existing predictors. This strategy also proposes that every pathogen protein is processed for cleavage sites, irrespective of subcellular location.

One uncertainty in the approach is that it is not clear whether the fragmentation of a protein via a host's (e.g., human) proteasome is the same each time. In other words, do we always end up with the same set of peptide fragments given the same pathogen? Another uncertainty

is the use of peptides in a multi-epitope vaccine. As an example, assume peptides are predicted that have a high binding affinity to MHC II molecules, irrespective of the prediction method. In the typical vaccinology workflow, these peptide sequences are linked together along with an adjuvant sequence to form a vaccine construct. Our expectation is that it is the adjuvant providing the pathogen-associated molecular patterns (PAMPs) (and not the peptide candidates) that are recognised by the APCs. In such a case, the vaccine construct once engulfed by the APCs would be fragmented. What is not clear is whether the intended peptide fragments would be retained. It is expected that they will not be because the original proteasomal cleavage sites on the parent proteins no longer exist in the vaccine construct. Our current leaning for obtaining a cell-mediated response is to include entire proteins in a vaccine formulation, and in particular proteins that contain a high density of peptides with high binding affinity. The expectation is that some of the desired proteolytically fragmented peptides will be presented for T-cell helper inspection.

Testing existing programs that predict proteasomal cleavage sites

Only one program could be found that predicts which peptides are naturally processed by the MHC class II antigen presenting pathway (there are more programs available for MHC I e.g., Netchop). The one MHC II program is called NetCleave and as quoted in the publication, "predictions achieve great predictive power towards class I isotypes (AUC ~ 0.92) and modest predictive power towards class II isotypes (AUC ~ 0.66)". Predictions of 66% for MHC II are discouraging because this poor accuracy will obviously have a detrimental knock-on effect on other *in silico* vaccine discovery steps.

For testing existing tools, known peptides were downloaded from IEDB (see Supplementary Tables for Data S1, sheet [IEDB_epitopes]). Disappointingly, there are only 23 peptides from 15 *Toxoplasma gondii* proteins (see below Appendix A: Test_analysis). And even some of these peptides are questionable e.g., the peptides are the outcome from 13 publications and five are more than 20 years old.

The only program that specifically predicted cleavage sites for MHC II ligands, given an antigen sequence, was MHCII-NP (see Appendix A: Test_analysis). This program, however, did not correctly predict any of the cleavage sites of the 23 *T. gondii* peptides (see Appendix B – MHCII-NP_results). It is unclear if the test peptides are correct or the program is simply inaccurate. As a further test, two human MHCII peptides from IEDB were tried, but the cleavage sites were still incorrectly predicted by MHCII-NP. MHCII-NP uses previously identified motifs from 10 residues prior and beyond the N- and C-terminuses of known peptides to predict the cleavage sites (no machine learning is used). NetCleave (developed in 2021) uses neural networks to predict C-terminal cleavage sites. However, it strangely does not take antigen sequences for the input, which seems to defeat the purpose of the predictor i.e., the input is only seven residues and it makes a prediction on the assumption that four residues occur before the cleavage site and three after. Furthermore, one needs to specify one of four MHC II allele classes (HLA, HLA_DP, HLA_DQ, HLA_DR) as part of the input. There are other recent programs like ITCell (see Appendix A: Test_analysis) that predict

cleavage sites as part of their strategy for predicting T-cell epitopes but do not show the cleavage sites in their results.

Developing a program to predict cleavage sites

It appears that current peptide-MHC binding predictors do not take in to account MHC ligands/fragments as a result of natural antigen processing. Moreover, it is not clear whether the lengths of amino acids ‘predicted’ as peptides exactly correspond to a fragment length generated by proteolytic break down of the protein.

Proposed methodology #1

Known MHC II peptides will be downloaded from IEDB. Ideally, the host organism for the antigen processing should be ‘human’ and the source of the antigen, an infectious organism.

Given the source antigen sequence of the peptide in the following context:

N-terminal sequence + peptide sequence + C-terminal sequence

Note: a MHCII peptide sequence is typically 12 to 25 amino acids (AAs) in length, but potentially can be an entire protein. Peptide binding to MHCII is mainly determined by interactions within a limited sized binding groove, which means a consecutive stretch of only 9 AAs of the peptide typically does the binding (i.e., the ‘binding core’). Residues protruding from either side of the binding groove are commonly known as **peptide flanking regions** (PFRs). Note that peptides are possibly trimmed for presentation after protein is fragmented, which may complicate predictions. The source antigen in the context of the above characteristics would be:

Variable N-terminal sequence + variable PFR + 9AAs binding core + variable PFR + variable C-terminal sequence

A proposed training data sequence for the N-terminal:

> 10AAs preceding peptide + first 3AAs from PFR

A proposed training data sequence for the C-terminal:

> Last 3AAs from PFR + 10AAs following peptide
>
> Note: This means that in the training data only peptides >= 15 are used (e.g. first 3AAs from PFR + variable number of AAs from PFR + 9 AAs binding core + last 3AAs from PFR + variable number of AAs from PFR)

Here is an example in machine learning (ML) terms (except letters will be converted to a pre-set digital value):

20 AA MHC II peptide = AQWEFQIGPCEGISMGDHLW

Antigen =
RACLYAGVKIAGTNAEVMPAQWEFQIGPCEGISMGDHLWVARFILHRVCEDFGVIA
TFDP

N-Terminal = IAGTNAEVMP + AQW

C-Terminal = HLW + VARFILHRVC

N-Terminal ML header (i.e., features):
ID,P1,P2,P3,P4,P5,P6,P7,P8,P9,P10,PFR1,PFR2,PFR3,Target

C-Terminal ML header: ID, PFR1,PFR2,PFR3,P1,P2,P3,P4,P5,P6,P7,P8,P9,P10,Target

For the negative training sequences, all the positive training sequences are used but randomly shuffled. Different ML algorithms and models will be trained and evaluated to see which one performs the best on test data. The expectation is that there will be one ML model for the N-terminal and one for the C-terminal cleavage site.

Here is an example of how the input sequences for predicting the cleavage sites are represented.

Example sequence:
MTTSASSHLNKGIKQVYMSLPQGEKVQAMYIWIDGTGEGLRCKTRTLDSEPKCVEELPEW

The sequence is broken up in accordance to a fixed sliding window of 13 AAs. Hence the sequences for the N-terminal cleavage sites model will be:

```
>N10
MTTSASSHLNKGI
>N11
TTSASSHLNKGIK
>N12
TSASSHLNKGIKQ
```

…and so on

The sequences for the C-terminal cleavage sites model will be:

```
>C3
MTTSASSHLNKGI
>C4
TTSASSHLNKGIK
>C5
TSASSHLNKGIKQ
```

…and so on

Following the predictions, there will be a probability score for the classification correctness of the sequences N10, N11, N12 etc. and C3, C4, C5 etc. Each sequence has a number associated with its identifier. This number represents the position in the antigen sequence where the cleavage is expected to occur.

The aim is to take a high-scoring N sequence and then find its nearest high-scoring C sequence. If the length between the N and the C (given their sequence identifiers) is >= 15 and =<25 then the sequence between the two sites will be flagged as a possible ligand.

Proposed methodology #2

The strongest motif signals in the vicinity of cleavage sites are in the PFR regions. An alternative method is to use different ML models for different peptide lengths e.g., one model trained, as above, on only peptides of length 15 and 16 (with 3AAs from PFR in the training sequence), and then another model for peptides of length 17 and 18 (with 4AAs from PFR in the training sequence), and then one for 19 and 20 (with 5AAs from PFR), and so on. This is still based on the assumption that the binding core is 9AAs.

The input sequences would be broken up as before but with a 13 AA fixed sliding window, then a 14AA window, then a 15AA, and so on. The different sets of sequence lengths from the same source antigen would be processed with the corresponding models e.g., 15-16 model, 17-18 model, and 19-20 model.

The length thresholds between the highest-scoring N and the C sites would therefore be ‘>14 and <17’, and ‘> 16 and <19’ for the 15-16 model and 17-18 model, respectively. The final outcome would be a list of ligands ranked on their cleavage site scores.

Results

Using the methodology #1 previously described, two training datasets representing over 50,000 MHC II peptides of length 15, one for the N- and the other for the C-terminal were created. The most frequent length for a MHC II peptide is 15 AAs (see Supplementary Tables for Data S1, sheet [Peptide Lengths 8 to 26 AAs]). The peptide lengths were compiled from 470,217 epitopes associated with 18,514 parent proteins downloaded from IEDB (See Appendix C: Predicting cleavage sites for more details**)**

Disappointingly, the ML prediction accuracy averaged only around 57% using 70% / 30% data split for testing. The algorithms used with default parameters were Random Forest, SVM, and neural networks using). One promising aspect is that there are clear signals to exploit. Refer to Supplementary Tables for Data S1, sheet [AA_frequencies]. This sheet shows the frequency of the 20 amino acids (AA) given 17,944 parent proteins of 52,976 peptides of length 15AAs e.g., 9.94% are ‘L’, 8.21% are ‘S’.

Sheet [N_L15_Pos_Context10_PFR3] shows in descending order the ratio of observed and expected frequencies at positions P1-P10 (the context) representing 10AA before the N-Terminal of the peptide, and positions PFR1-3 representing the flanking regions of the core binding region (typically 9AAs). For example, a ratio of 1.0 is exactly the frequencies you would expect by chance at a specific position. AAs at positions that are well above expectation are denoted by +++ or below expectation by ---). One can clearly see that the most informative positions are P10 (the expected cleavage site) and the flanking regions. To support these finding [N_L15_Neg_Context10_PFR3] shows the frequencies if the AAs in the 52,976 peptides are randomly shuffled. Similar results for the C-Terminal end were observed (see sheets [C_L15_Pos_PFR3_Context10] and [C_L15_Neg_PFR3_Context10]. To show that there are no informative positions before P1-P10, the context was changed to 20 (see sheet [N_L15_Pos_Context20 PFR9]).

The challenge is that although there appears to be signals at P10 and PFR1-3, there is also a huge amount of noise. Take PFR2 for example in sheet [N_L15_Pos_Context10_PFR3]. For a completely noiseless signal all 52,976 peptides at PFR2 would have the same AA e.g., ‘P’. However, all 20 AAs are present at PFR2, albeit at different frequencies. Nonetheless, the ratio (observed/expected) suggests that P is present at this location not by chance. The expected number of Ps at position PFR2 is 3,311 but we observe 5,467. Our challenge is that there is no clear motif. That is, although P is the most frequent AA at PFR2, only 10.3% of the peptides in our training data have P at this location (7.8% for A, 7.6% G etc.)

The expected reason for only obtaining 57% accuracy from the ML models is the excessive noise e.g., 93.2% accuracy can be achieved if ‘P’ is the only AA at PFR2 (100% accuracy was not obtained because some negative peptides also have a P at PFR2). This latter high accuracy supports that the default ML parameters, the number of features, and the methodology used are fit for the purpose

Further analysis of the training data is required. For example, it may be informative to determine how many of the 52,976 peptides have different AAs to that expected at P10 and PFR1-3. Then analyse whether there are other informative differences (other than AAs) between those ‘meeting’ and those ‘not meeting’ expectations. Maybe above or below expectation values can be used as predictors in the training data. Also, it may be worth investigating whether there is any chemical and/or physical commonality between the most frequent AAs that do not meet expectations e.g., are the AA frequencies that exceed expectation hydrophilic? Then maybe chemical and physical properties could be used for the predictors.

The following ML predictor types for the context and flanking regions of MHCII peptides were tested using 70% / 30% data split of the training data, but the ML prediction accuracy averaged only around 57%

- Amino acids e.g., P1 = 15 (for C), P2= 20 (for P)
- Ratios (observed/expected) e.g., P1 = 0.974 (for L), P2 = 0.888 (for S)
- AA property types (i.e., grouping amino acids on properties such as Charge, Polar, Amphipathic, and Hydrophobic) e.g., P1 = 1 (for Charged R K D E), P2 = 4 (for Hydrophobic A I L F V P G) and also grouping amino acids based on R Groups

The source for the training data was reanalysed. In IEDB, there are 627,721 MHC II related ligands (peptides) associated with 687 publications. The publication years range from 1989 to 2022. The question is how reliable are these peptides? How can we determine which of the 627,721 peptides are bona fide peptides? The expectation is that peptides determined in 1989 would not be as accurate as those from 2022.

The above 70%/30% tests were repeated but only with peptides determined in the last 10 years (13,333 peptides). The results improved slightly but still averaged only around 60% accuracy. It is still debatable, however, whether the slight improvement was gained by weeding out possible inaccurate peptides.

Given the the13,333 peptides, many peptides are associated with one or more publications in. For example, one peptide from the same protein is associated with 20 publications (the highest noted association); and 1,910 (14.3%) peptides are associated with only one publication (the lowest noted association). Our assumption is that peptides associated with several publications are possibly more reliable than those with fewer publications. With this in mind, the 13,333 dataset was weighted as follows: each peptide was duplicated according to the number of associated publications e.g., the peptide associated with the 20 publications was duplicated so that a further 19 identical peptides were added to the 13,333; for peptides associated with 19 publications, 18 identical peptides were added to the total and so.

The above 70%/30% tests were repeated and a well over 90% accuracy was obtained. However, our approach of weighting the data may possibly be too subjective and needs to be evaluated by machine learning experts. The results have, however, raised some interesting points. For example, each group of identical peptides represent less than 1% of the total number, but collectively the introduction of these identical peptides clearly boosts the signal for ML. Furthermore, the context and flanking regions of the 13,333 peptides were clustered based on sequence similarity. Based on 40% similarity, 103 clusters (groups) were generated with 497 members in the largest group and only three in the smallest. If the 70%/30% tests are performed using the members from each group to create 103 training models (i.e., the positive dataset only contains the number of members e.g., 497 will be the largest training dataset), the accuracy is over 80% for the larger membership models. This is not proposed as a solution, but it seems to support the idea that there is no universal cleavage signal and that there are possible natural groups of peptides.

All the Literature was note fully investigated on cleavage site prediction from the endocytic proteases' perceptive, it seems to suggest, "the three main classes of intracellular proteases residing in the lysosomal/endosomal compartments and participating in antigen degradation are 1) cysteine (cathepsin B, F, H, L, S, Z, and AEP, for asparaginylendopeptidase); 2) aspartate (cathepsin D, E); and 3) serine (cathepsin A, G) proteases". The latter statement implies that there should be three main groups of peptides distinguished by the amino acids C, D, and S, respectively in the vicinity of the natural cleavage site. Unfortunately, this was not the case when the 627,721 peptides from IEDB were analysed, and IEDB does not make any association between proteases and peptides. Elsewhere in the Literature it also states, "Most endocytic proteases display broad cleavage specificity". Although this seems to be a contradiction to the previously described three classes of intracellular proteases, we believe the way forward is to find the 'appropriate' grouping of the source peptides to create separate training models e.g., searching for amino acid cleavage motifs for each cathepsin class. The aim would be to create an ML model for each class. From our searching so far, there appears to be no record in any peptide database of which cathepsin contributed to the known peptide.

Appendixes

Appendix A: Text analysis

<u>Test data</u>

Downloaded from IEDB:

34 linear peptides known to be MHC II ligands based on 70 assays associated with 13 publications from 1991 to 2018 (5 of the 13 are more than 20 years old).

The 34 peptides are sourced from 20 parent proteins from *Toxoplasma gondii*

The host organism in the 70 assays:

> Homo sapiens (human), Mus musculus (mouse), Mus musculus BALB/c, Mus musculus C57BL/10 X C3H/HeJ, Mus musculus C57BL/6, Mus musculus CBA/J, Rattus norvegicus Fischer

Allele names associated with the 70 assays:

HLA-DPw4, HLA class II, HLA-DR4, H2-d class II, H2-k class II, H2-IAb, H2-b class II, RT1 class II

5 parent proteins are no longer valid and therefore there are only 23 peptides from 15 proteins available for testing.

MHCII-NP (2018)

Source: http://tools.iedb.org/mhciinp/

Online program to predict which MHC II ligands are generated as a result of **natural antigen processing**.

Dataset:

14,000 naturally processed ligands identified by mass spectrometry of peptides eluted from MHC class II expressing. Data downloaded from IEDB. It is not clear which organism was the host for the antigen processing. I think that it should be only be one host e.g., human,

Methodology:

Identify sequence signatures potentially related to the cleavage mechanisms that liberate the presented peptides from their source antigens (no machine learning used)

The sequence regions analysed included 10 residues prior and past the N- and C-terminuses of each ligand including residues at both termini.

NetCleave (2021)

Source: https://github.com/pepamengual/NetCleave

A Standalone program that predicts the C-terminal antigen processing for both MHC-I and MHC-II pathways.

Methodology:

Uses a neural network trained on 46 different physicochemical descriptors of the cleavage site amino acids

Uses a short sequence of seven residues to generate predictions: four residues placed before and three after the cleavage site. Each residue is encoded with 46 amino acid descriptors (describing steric, electrostatic and hydrophobic properties), resulting in a total amount of 336 descriptors for each short sequence i.e., 7aa * 46 = 322?? Extra 14?

ITCell (Integrative T-cell epitope prediction) (2018)

Source: http://salilab.org/itcell

This online program predicts cleavage sites, peptide-MHC II binding, and TCR epitope recognition. The output does not display the cleavage site location

The program requires the user to specify the MHC type or upload their own peptide-MHC structure (or model) in PDB format AND the TCR structure in a PDB format i.e., it predicts T-cell epitopes, given the antigen, MHCII, and TCR sequences.

Methodology for cleavage sites:

Cleavage sites are predicted in the antigen sequence based on cathepsins S, B, and H cleavage profiles. These sites are used to filter the set of all possible 12-mer peptides for the next step (peptide-MHCII predictions).

Cathepsin S has a preference for hydrophobic residues in the P2 position and for positively charged residues in the P1 position, consistent with the profiling of the P1-P4 positions using a combinatorial library. The remaining positions have broad specificity, consistent with the major role of this enzyme in antigen processing.

The cleavage site predictor was built for each cathepsin individually. First, the MSP-MS results were summarized in a matrix that contained amino acid residue counts from the cleaved peptides for each of the eight positions (P4 to P4’). This matrix was then used to compute a Hidden Markov Model (HMM) for cleavage prediction. The residue counts were converted to probabilities, normalized by the natural occurrence of amino acid residues; and the score, cleavage_score, is the sum of the log of normalized probabilities. All octapeptides in the antigen sequence are considered for proteolytic cleavage at the P1-P1’position. For a confident cleavage prediction, we require cleavage_score > 3.0, corresponding to at least three times higher likelihood of cleavage than by chance. We first consider cleavage by endopeptidases cathepsins B and S, followed by the aminopeptidase cathepsin H for additional trimming at the N-terminus; we do not use the cathepsin H profile for endoprotease cleavage, because our specificity matrix was constructed using only aminopeptidase cleavage sites.

Questions:

Is the proteolytic break down of a specific protein always the same each time i.e., do we always end up with the same number of peptides with the same sequence?

Is the breakdown different for different host organisms e.g., given the same protein, end up with a different set of peptides in a cow as opposed to a human?

Is the breakdown different for different populations of humans or individuals?

Are peptides trimmed for presentation after protein is fragmented?

Notes:

In the main endocytic pathway of antigen presentation, extracellular antigens are internalized by phagocytosis, macropinocytosis, or receptor-mediated endocytosis, and degraded in acidic and proteolytic compartments such as lysosomes or late endosomes by proteases generally called cathepsins.

Factors beyond MHC class II binding affinity contribute to likelihood of a peptide to be recognized by CD4+ T cells i.e., predicting an MHCII binding peptide does not always equate to a conclusive prediction of a T-cell epitope.

It is widely recognized that natural processing shapes which peptides are available for binding to MHC molecules and subsequently presented to T cells, and that the capacity to bind MHC molecule is a necessary but not sufficient requisite for immunogenicity.

Appendix B – MHCII-NP_results

>sp|P13664|P30_TOXGO Major surface antigen p30 OS=Toxoplasma gondii OX=5811 PE=1 SV=1

MSVSLHHFIISSGFLTSMFPKAVRRAVTAGVFAAPTLMSFLRCGVMASDPPLVANQVVTC

PDKKSTAAVILTPTENHFTLKCPKTALTEPPTLAYSPNRQICPAGTTSSCTSKAVTLSSL

IPEAEDSWWTGDSASLDTAGIKLTVPIEKFPVTTQTFVVGCIKGDDAQSCMVTVTVQARA

SSVVNNVARCSYGADSTLGPVKLSAEGPTTMTLVCGKDGVKVPQDNNQYCSGTTLTGCNE

KSFKDILPKLTENPWQGNASSDKGATLTIKKEAFPAESKSVIIGCTGGSPEKHHCTVKLE

FAGAAGSAKSAAGTASHVSIFAMVIGLIGSIAACVA

Known epitopes from IEDB

**SDPPLVANQVVTCPDKKSTA**	**48**	**67**	**20**	
**CPKTALTEPPTLAYSPNRQIC**	**82**	**102**	**21**	
**VTVTVQARASSVVNNV**		**172**	**187**	**16**
**NNVARCSYGADSTLGPV**		**185**	**201**	**17**
**GPVKLSAEGPTTMTLV**		**199**	**214**	**16**
**LVCGKDGVKVPQDNNQYC**		**213**	**230**	**18**
**CNEKSFKDILPKLTENPWQ**		**238**	**256**	**19**
**KSVIIGCTGGSPEKHHC**		**279**	**295**	**17**
**FAGAAGSAKSAAGTASHVSI**	**301**	**320**	**20**	

MHCII-NP: Prediction of naturally processed MHC II ligands

Seq name	Peptide start	Peptide end	Peptide length	Peptide	N motif	C motif	Cleavage probability score	Cleavage probability percentile rank
5 SP|P13664|P30_TOXGO MAJOR SURFACE ANTIGEN P30 ...	49	62	14	DPPLVANQVVTCPD	SDP	PDK	1.04094	0.11
1 SP|P13664|P30_TOXGO MAJOR SURFACE ANTIGEN P30 ...	49	63	15	DPPLVANQVVTCPDK	SDP	DKK	1.47081	0.00
3 SP|P13664|P30_TOXGO MAJOR SURFACE ANTIGEN P30 ...	49	64	16	DPPLVANQVVTCPDKK	SDP	KKS	1.26165	0.06
4 SP|P13664|P30_TOXGO MAJOR SURFACE ANTIGEN P30 ...	89	104	16	EPPTLAYSPNRQICPA	TEP	PAG	1.22400	0.09
2 SP|P13664|P30_TOXGO MAJOR SURFACE ANTIGEN P30 ...	207	221	15	GPTTMTLVCGKDGVK	EGP	VKV	1.35743	0.03

AES KSVIIGCTGGSPEKHHC TVK

>tr|Q5UAF5|Q5UAF5_TOXGO Dense granular protein GRA10 OS=Toxoplasma gondii OX=5811 PE=2 SV=1

MIEAAVEAAKYEEVLACIKDYAQFGSRPAEPWQHPASQTSSAAAATPGLPKKGVPVGGIG

QEGSRVPPPPPPPPGSVGESGFSLSSGSGVSVVEKRDVSSEGRGPSDGVSISFNARGASL

PGSLGDERKALETGVFGIPVSLGPPSLGLRGDPGEPSNPYLGYCALLPLLTEEQFRHIRR

LQKRAMVLCGYTQSPPESRKKRRRSGKKKRGKRSVSSHSTGSGTLPSDEPVDGCDRVREE

AEREGTGCMSADAPAVDTNTEALSIQNAATSVTRQPLSDPDSANATGSRVRAGRRALPRV

PPVLKISRIGDKNDTDTTQNKDTGSTQSQRANSVIETCSVVEENTMAKIPGAEAKPEGGG

ETPV

Known epitopes from IEDB

SGFSLSSGSGVSVVE 80 94 15

MHCII-NP: Prediction of naturally processed MHC II ligands

Seq name	Peptide start	Peptide end	Peptide length	Peptide	N motif	C motif	Cleavage probability score	Cleavage probability percentile rank
1 TR|Q5UAF5|Q5UAF5_TOXGO DENSE GRANULAR PROTEIN ...	155	169	15	EPSNPYLGYCALLPL	GEP	PLL	1.35314	0.00
2 TR|Q5UAF5|Q5UAF5_TOXGO DENSE GRANULAR PROTEIN ...	143	157	15	GPPSLGLRGDPGEPS	LGP	PSN	1.12579	0.03
3 TR|Q5UAF5|Q5UAF5_TOXGO DENSE GRANULAR PROTEIN ...	279	293	15	DPDSANATGSRVRAG	SDP	AGR	0.99029	0.05
4 TR|Q5UAF5|Q5UAF5_TOXGO DENSE GRANULAR PROTEIN ...	229	243	15	EPVDGCDRVREEAER	DEP	ERE	0.96172	0.08
5 TR|Q5UAF5|Q5UAF5_TOXGO DENSE GRANULAR PROTEIN ...	34	48	15	HPASQTSSAAAATPG	QHP	PGL	0.95090	0.11

>tr|A0A086KD61|A0A086KD61_TOXGO Dense granule protein GRA2 OS=Toxoplasma gondii p89 OX=943119 GN=TGP89_227620 PE=4 SV=1

MFAVKHCLLVVAVGALVNVSVRAAEFSGVVNQGPVDVPFSGKPLDERAVGGKGEHTPPLP

DERQQEPEEPVSQRASRVAEQLFRKFLKFAENVGQHSEKAFKKAKVVAEKGFTAAKTHTV

RGFKVAKEAAGRGMVTVGKKLANVESDRSTTTTQAPDSPNGLAETEVPVEPQQRAAHVPV

PDFSQ

Known epitopes from IEDB

RASRVAEQLFRKFLKF	74	89	16

MHCII-NP: Prediction of naturally processed MHC II ligands

	Seq name	Peptide start	Peptide end	Peptide length	Peptide	N motif	C motif	Cleavage probability score	Cleavage probability percentile rank
1	TR|A0A086KD61|A0A086KD61_TOXGO DENSE GRANULE P...	167	181	15	VPVEPQQRAAHVPVP	EVP	VPD	1.41145	0.00
2	TR|A0A086KD61|A0A086KD61_TOXGO DENSE GRANULE P...	33	47	15	GPVDVPFSGKPLDER	QGP	ERA	1.33087	0.05
3	TR|A0A086KD61|A0A086KD61_TOXGO DENSE GRANULE P...	66	79	14	EPEEPVSQRASRVA	QEP	VAE	1.28624	0.11
4	TR|A0A086KD61|A0A086KD61_TOXGO DENSE GRANULE P...	155	168	14	APDSPNGLAETEVP	QAP	VPV	1.23310	0.16
5	TR|A0A086KD61|A0A086KD61_TOXGO DENSE GRANULE P...	69	84	16	EPVSQRASRVAEQLFR	EEP	FRK	1.22400	0.22

>tr|A0A086JP35|A0A086JP35_TOXGO Bradyzoite antigen BAG1 OS=Toxoplasma gondii GAB2-2007-GAL-DOM2 OX=1130820 GN=TGDOM2_259020 PE=3 SV=1

MAPSASHPPGACPPGCTKHPATATAISPSGVCPMRAFHPAGSHSHFSCYDDLRNRLSHDK

NVRPVASQQLDYLDEVSPFALAYYPPPFWGGVGLNPIDDMLFETALTANEMMEDITWRPR

VDVEFDSKKKEMIILADLPGLQKDDVTIEVDNGAIVIKGEKTSKEAEKVDDGKTKNILTE

RVSGYFARRFQLPSNYKPDGISAAMDNGVLRVTIKVEDSGGAKQQISVK

Known epitopes from IEDB

GYFARRFQL	184	192	9

Seq name	Peptide start	Peptide end	Peptide length	Peptide	N motif	C motif	Cleavage probability score	Cleavage probability percentile rank

1 TR|A0A086JP35|A0A086JP35_TOXGO BRADYZOITE ANTI... 2 15 14
APSASHPPGACPPG MAP PGC 1.26450 0.00

2 TR|A0A086JP35|A0A086JP35_TOXGO BRADYZOITE ANTI... 126 140 15
DSKKKEMIILADLPG FDS PGL 1.00811 0.04

3 TR|A0A086JP35|A0A086JP35_TOXGO BRADYZOITE ANTI... 197 211 15
KPDGISAAMDNGVLR YKP LRV 0.85835 0.09

4 TR|A0A086JP35|A0A086JP35_TOXGO BRADYZOITE ANTI... 113 128 16
EDITWRPRVDVEFDSK MED SKK 0.82066 0.13

5 TR|A0A086JP35|A0A086JP35_TOXGO BRADYZOITE ANTI... 2 14 13
APSASHPPGACPP MAP PPG 0.76804 0.17

>tr|B9Q5Z2|B9Q5Z2_TOXGV Dense granule protein 1 / major antigenp24 OS=Toxoplasma gondii (strain ATCC 50861 / VEG) OX=432359 GN=BN1205_017550 PE=4 SV=1

MVRVSAIVGAAASVFVCLSAGAYAAEGGDNQSSAVSDRASLLGLLSGGTGQGLGIGESVD

LEMMGNTYRVERPTGNPDLLKIAIKTSDGSYSEVGDVNVEEVIDTMKSMQRDEDIFLRAL

NKGETVEEAIEDVAQAEGLNSEQTLQLEDAVSAVASVVQDEMKVIDDVQQLEKDKQQLKD

DIGFLTGERE

Known epitopes from IEDB

CSLKKSSKMVRVSAIV

Epitope starts before protein?

MVRVSAIVGAAASVFVCLS

CSLKKSSKMVRVSAIV

Seq name Peptide start Peptide end Peptide length Peptide N motif C motif Cleavage probability score Cleavage probability percentile rank

1 TR|B9Q5Z2|B9Q5Z2_TOXGV DENSE GRANULE PROTEIN 1... 29 43 15
DNQSSAVSDRASLLG GDN LGL 0.90725 0.00

2 TR|B9Q5Z2|B9Q5Z2_TOXGV DENSE GRANULE PROTEIN 1... 72 85 14
RPTGNPDLLKIAIK ERP IKT 0.86363 0.05

3 TR|B9Q5Z2|B9Q5Z2_TOXGV DENSE GRANULE PROTEIN 1... 28 43 16
GDNQSSAVSDRASLLG GGD LGL 0.82066 0.11

4 TR|B9Q5Z2|B9Q5Z2_TOXGV DENSE GRANULE PROTEIN 1... 65 78 14
GNTYRVERPTGNPD MGN PDL 0.66015 0.16

5 TR|B9Q5Z2|B9Q5Z2_TOXGV DENSE GRANULE PROTEIN 1... 160 175 16
DEMKVIDDVQQLEKDK QDE DKQ 0.59986 0.21

>tr|A0SIX7|A0SIX7_TOXGO Dense granule antigen OS=Toxoplasma gondii OX=5811 GN=GRA7 PE=2 SV=1

MARHAIFSALCVLGLVAAALPQFATAATASDDELMSRIRNSDFFDGQAPVDSLRPTNAGV

DSKGTDDHLTTSMDKASVESQLPRREPLETEPDEQEEVHFRKRGVRSDAEVTDDNIYEEH

TDRKVVPRKSEGKRSFKDLLKKLALPAVGMGASYFAADRLVPELTEEQQRGDEPLTTGQN

VGTVLGFAALAAAAAFLGMGLTRTYRHFSPRKNRSRQPALEQEVPESGEDGEDARQ

Known epitopes from IEDB

ALAAAAAFL	189	197	9

Seq name Peptide start Peptide end Peptide length Peptide N motif C motif Cleavage probability score Cleavage probability percentile rank

Seq name		Peptide start	Peptide end	Peptide length	Peptide	N motif	C motif	Cleavage probability score	Cleavage probability percentile rank
1	TR|A0SIX7|A0SIX7_TOXGO DENSE GRANULE ANTIGEN O...	86	101	16	EPLETEPDEQEEVHFR	REP	FRK	1.20953	0.00
2	TR|A0SIX7|A0SIX7_TOXGO DENSE GRANULE ANTIGEN O...	91	104	14	EPDEQEEVHFRKRG	TEP	RGV	1.04791	0.04
3	TR|A0SIX7|A0SIX7_TOXGO DENSE GRANULE ANTIGEN O...	91	106	16	EPDEQEEVHFRKRGVR	TEP	VRS	1.04562	0.08
4	TR|A0SIX7|A0SIX7_TOXGO DENSE GRANULE ANTIGEN O...	48	61	14	APVDSLRPTNAGVD	QAP	VDS	0.96643	0.13
5	TR|A0SIX7|A0SIX7_TOXGO DENSE GRANULE ANTIGEN O...	209	223	15	SPRKNRSRQPALEQE		FSP	QEV	0.93919 0.17

>tr|A0A125YNH9|A0A125YNH9_TOXGG Uncharacterized protein OS=Toxoplasma gondii (strain ATCC 50853 / GT1) OX=507601 GN=TGGT1_212300 PE=4 SV=1

MSAQGQQGVRSTVAAGKNRFWQPAPITVLLVLSSSFLFAFAANTPQNSSTTGQSTPSDDH

GVSTEHFHDAVTVDAAEHAGEDEMTACSPEAARVIEARVATLKAETEAAVAEVRRTEAMQ

RELFGRLNAKVRAMNDRLDELSDAQKSLAKRLETTGQLLIADREEQAKRFTTDVETYIIE

SGLKKAVFQQREIAAKLQTLAESNQKSKQALDALLQHVDDEKEQTKQWISTVVRDLEKYV

EARTQTTLESLQRDAQEHANFMQQQLNDMKLDHLLLLQAVSSAETQLNERGIAGVDISQL

DESASFERMMTTFEKVLKEQLKGKFVLPIFVVTQCPDSMERVAFMNTYLVPSKRNFRAVF

QALGHTPQASAVVIRIESVMVEAWMEVNGKVTGMQYLTPPAHEALTVTTKDRFGFEFLRR

VRELETGDFKADPHGRSVVIAFVGNRKGRIGGLPLTDVTEFNKFQALRTLATMHSSPIKE

TNNLWQIMKTEVDIVATFAAMPRVEVGGSSLRIYQGIAPEPRLPASPFCRNAFTLIRIEV

AKSPRGKQEYVYPKASLLKKVFDILVSTNTSQDSGGDGSFAVIRANVSNAGWLQSKLYAT

ADRMAVEIHRPVPGTAPPSFSSEDVAGEHRANSMAAVVDQIRKQMTDVVLPNIDPEDSTS

RRQVLVHICEIGVTCNSDRHPEA

Known epitopes from IEDB

AVEIHRPVPGTAPPS	605	619	16

Seq name		Peptide start	Peptide end	Peptide length	Peptide	N motif	C motif	Cleavage probability score	Cleavage probability percentile rank
1	TR|A0A125YNH9|A0A125YNH9_TOXGG UNCHARACTERIZED...	616	630	15	APPSFSSEDVAGEHR	TAP	HRA	1.21632	0.00

2 TR|A0A125YNH9|A0A125YNH9_TOXGG UNCHARACTERIZED... 432 446 15
DPHGRSVVIAFVGNR ADP NRK 1.07615 0.01

3 TR|A0A125YNH9|A0A125YNH9_TOXGG UNCHARACTERIZED... 523 537 15
LPASPFCRNAFTLIR RLP IRI 1.04022 0.03

4 TR|A0A125YNH9|A0A125YNH9_TOXGG UNCHARACTERIZED... 82 96 15
DEMTACSPEAARVIE EDE IEA 0.95672 0.04

5 TR|A0A125YNH9|A0A125YNH9_TOXGG UNCHARACTERIZED... 616 629 14
APPSFSSEDVAGEH TAP EHR 0.88421 0.05

>tr|S7V482|S7V482_TOXGG MAG1 protein OS=Toxoplasma gondii (strain ATCC 50853 / GT1) OX=507601 GN=MAG1 PE=4 SV=1

MDCGQCRRQLHAAGVLGLFVTLATATVGLSQRVPELPEVESFDEVGTGARRSGSIATLLP

QDAVLYENSEDVAVPSDSASTPSYFHVESPSASVEAATGAVGEVVPDCEERQEQGDTTLS

DHDFHSGGTEQEGLPETEVAHQHETEEQYGTEGMPPPVLPPAPVVHPRFIAVPGPSVPVP

FFSLPDIHPDQVVYILRVQGSGDFDISFEVGRAVKQLEAIKKAYREATGKLEADELESER

GPAVSPRRRLVDLIKDNQRRLRAALQKIKIQKKLEEIDDLLQLTRALKAMDARLRACQDM

APIEEALCHKTKAFGEMVSQKAKEIREKAASLSSLLGVDAVEKELRRVEPEHEDNTRVEA

RVEELQKALEKAASEAKQLVGTAAGEIEEGVKADTQAVQDSSKDVLTKSQLALVEAFKAI

QRALLEAKTKELVEPTSKEAEEARQILAEQAA

Known epitopes from IEDB

SASTPSYFH 78 86 9

Seq name Peptide start Peptide end Peptide length Peptide N motif C motif Cleavage probability score Cleavage probability percentile rank

1 TR|S7V482|S7V482_TOXGG MAG1 PROTEIN OS=TOXOPLA... 241 255 15
GPAVSPRRRLVDLIK RGP IKD 1.47081 0.00

2 TR|S7V482|S7V482_TOXGG MAG1 PROTEIN OS=TOXOPLA... 172 186 15
VPGPSVPVPFFSLPD AVP PDI 1.14287 0.02

3 TR|S7V482|S7V482_TOXGG MAG1 PROTEIN OS=TOXOPLA... 177 191 15
VPVPFFSLPDIHPDQ SVP DQV 1.06034 0.04

4 TR|S7V482|S7V482_TOXGG MAG1 PROTEIN OS=TOXOPLA... 177 190 14
VPVPFFSLPDIHPD SVP PDQ 1.05340 0.06

5 TR|S7V482|S7V482_TOXGG MAG1 PROTEIN OS=TOXOPLA... 154 168 15
MPPPVLPPAPVVHPR GMP PRF 0.98028 0.08

>sp|Q9XYH7|MIC6_TOXGO Micronemal protein 6 OS=Toxoplasma gondii OX=5811 GN=MIC6 PE=1 SV=1

MRLFRCCAAAVVAAESLLWLKNGSPFFAFLPGNGEIADNCSGNPCGGTAAGTCINTPSGY

DCRCEPGYVLGVENDQVTCMMPSGVPMANFVQLSETPAACSSNPCGPEAAGTCKETNSGY

ICRCNQGYRISLDGTGNVTCIVRQESGCEENGCGPPDAVQSCRRLTGTAGRLCVCKENFI

ATIDASAHITCKRVPPHYRKPPFEFGKGGHPVDSEPSKRQREDEGESREPESDSTEPGRD

QERRTPLEESQEPEGSTPDSQQSRGGSGSDSTESEEQGKEREEGSGHAGAIAGGVIGGLL

LLSAAGAGVAYMRKSGSGGGEEIEYERGIEAAEASEVEVLVDLDSKTWD

Known epitopes from IEDB

LLSAAGAGV 301 309 9

	Seq name	Peptide start	Peptide end	Peptide length	Peptide	N motif	C motif	Cleavage probability score	Cleavage probability percentile rank
1	SP|Q9XYH7|MIC6_TOXGO MICRONEMAL PROTEIN 6 OS=T...	229	243	15	EPESDSTEPGRDQER	REP	ERR	1.33087	0.00
2	SP|Q9XYH7|MIC6_TOXGO MICRONEMAL PROTEIN 6 OS=T...	85	98	14	VPMANFVQLSETPA	GVP	PAA	1.22732	0.03
3	SP|Q9XYH7|MIC6_TOXGO MICRONEMAL PROTEIN 6 OS=T...	229	244	16	EPESDSTEPGRDQERR	REP	RRT	1.20953	0.06
4	SP|Q9XYH7|MIC6_TOXGO MICRONEMAL PROTEIN 6 OS=T...	24	38	15	SPFFAFLPGNGEIAD	GSP	ADN	1.08902	0.08
5	SP|Q9XYH7|MIC6_TOXGO MICRONEMAL PROTEIN 6 OS=T...	223	238	16	DEGESREPESDSTEPG	EDE	PGR	1.02224	0.11

>tr|Q9GV96|Q9GV96_TOXGO p36 protein OS=Toxoplasma gondii OX=5811 GN=p36 PE=2 SV=1

MSSSNFRVGQMTHPNPLQTRFSRSASLVSRMPLVRLFFTIAAPLLFSPSPFPFLPLKTHC

LAIQLGKPQGSPPASQKEAIRDTGVSHQKGEPSDSSSEPKPQGTVAETPGAASAAAAEVG

RPSRSSAGPGKKKRGPSLFTEETMGPSKRHPCLKTLAAAVERFQAQHLTGRDAAKAFVDA

IQECGIQVVASDYDRTAISVHSGGSARRDDLSVLGALTPDFKLLGEELTRRNIPIYFVTF

SDKGENRGDRIAAGPLVEATLKASNANFEAQGVFGYYPPFYSEPEDYAPLGLSAPMPTDK

SFHIQQVSKASGVSEDKILLLDDDRANCVNFCRSGGAAIHVSGHEGFDFGAVRVVVKPSL

IMQ

Known epitopes from IEDB

VAETPGAASAAA 105 116 12

	Seq name	Peptide start	Peptide end	Peptide length	Peptide	N motif	C motif	Cleavage probability score	Cleavage probability percentile rank
1	TR|Q9GV96|Q9GV96_TOXGO P36 PROTEIN OS=TOXOPLAS...	91	106	16	EPSDSSSEPKPQGTVA	GEP	VAE	1.27674	0.00
2	TR|Q9GV96|Q9GV96_TOXGO P36 PROTEIN OS=TOXOPLAS...	277	291	15	YPPFYSEPEDYAPLG	YYP	LGL	1.17430	0.03
3	TR|Q9GV96|Q9GV96_TOXGO P36 PROTEIN OS=TOXOPLAS...	98	110	13	EPKPQGTVAETPG	SEP	PGA	1.11367	0.05

4 TR|Q9GV96|Q9GV96_TOXGO P36 PROTEIN OS=TOXOPLAS... 254 267 14
GPLVEATLKASNAN AGP ANF 1.04791 0.08

5 TR|Q9GV96|Q9GV96_TOXGO P36 PROTEIN OS=TOXOPLAS... 288 303 16
APLGLSAPMPTDKSFH YAP FHI 0.98509 0.11

>tr|I7CQ43|I7CQ43_TOXGO Rhoptry kinase family protein OS=Toxoplasma gondii OX=5811 GN=ROP18 PE=4 SV=1

MFSVQRPSLTCTVVRMGLATLLRKTACLAGLTVALVFLLFQVQDGTGITLCPSKLDSKPT

ISLDSQQHVANKRGSATVGHYKYSLAGATESTRDVSLLEERAQHGVNTQETNQRRTTFQR

LVNRLRRRERDGAVSGFAADSPSRPRLSVRQRLAQLLRRAKSFFTHGIRRYYSQGRNRLR

SWWAQRRRSGLVFEKADSGCVIGKRILAHMREQIRQPQALESSQRLDGILTAAAWPPDVP

AKFVSLTTGETRTLVRGAPLGSGGFAAVYQVTDVETNEELAVKVIISEKKPTDETMRDLD

RESFCYKNFSLAKTAKDAQENWRFMVASDVVTLEGQPATTEVVIGSATKWVPNYFLLMMR

AETDMSKVISWLFGDASVNNSELGLVVRMHLSSQAIRLVANVQAQGIVHTDIKPPNFLLL

KDGRLFLGDFGTYKINNSVGPAIGTPGYEPPEQPFHSYGVTYTFATDAWQLGITLYCIWC

KERPTPADGIWDYLHFADCSSTPELVQDLIRNLLNREPQKRMLPLQALKTAAFNEMDSVV

KRAAQNFEQQEHLQTE

Known epitopes from IEDB

PPEQPFHSYGVTYTFATDA 450 468 19

Seq name Peptide start Peptide end Peptide length Peptide N motif C motif Cleavage probability score Cleavage probability percentile rank

1 TR|I7CQ43|I7CQ43_TOXGO RHOPTRY KINASE FAMILY P... 141 155 15
SPSRPRLSVRQRLAQ DSP AQL 1.14959 0.00

2 TR|I7CQ43|I7CQ43_TOXGO RHOPTRY KINASE FAMILY P... 131 146 16
DGAVSGFAADSPSRPR RDG PRL 0.92236 0.02

3 TR|I7CQ43|I7CQ43_TOXGO RHOPTRY KINASE FAMILY P... 374 388 15
GDASVNNSELGLVVR FGD VRM 0.90259 0.03

4 TR|I7CQ43|I7CQ43_TOXGO RHOPTRY KINASE FAMILY P... 144 159 16
RPRLSVRQRLAQLLRR SRP RRA 0.80760 0.05

5 TR|I7CQ43|I7CQ43_TOXGO RHOPTRY KINASE FAMILY P... 351 364 14
VPNYFLLMMRAETD WVP TDM 0.79993 0.07

>tr|Q2PAY2|Q2PAY2_TOXGO Rhoptry kinase family protein OS=Toxoplasma gondii OX=5811 GN=rop18 PE=1 SV=1

MFSVQRPPLTRTVVRMGLATLLPKTACLAGLNVALVFLLFQVQDGTGITLGPSKLDSKPT

SLDSQQHVADKRWLATVGHYKHLAGATESTRDVSLLEERAQHRVNAQETNQRRTIFQRLL

NLLRRRERDGEVSGSAADSSSRPRLSVRQRLAQLWRRAKSLFKRGIRRYFPQGRNRQRSL

RAQRRRSELVFEKADSGCVIGKRILAHMQEQIGQPQALENSERLDRILTVAAWPPDVPKR

FVSVTTGETRTLVRGAPLGSGGFATVYEATDVETNEELAVKVFMSEKEPTDETMLDLQRE

SSCYRNFSLAKTAKDAQESCRFMVPSDVVMLEGQPASTEVVIGLTTRWVPNYFLLMMRAE

ADMSKVISWVFGDASVNKSEFGLVVRMYLSSQAIKLVANVQAQGIVHTDIKPANFLLLKD

GRLFLGDFGTYRINNSVGRAIGTPGYEPPERPFQATGITYTFPTDAWQLGITLYCIWCKE

RPTPADGIWDYLHFADCPSTPELVQDLIRSLLNRDPQKRMLPLQALETAAFKEMDSVVKG

AAQNFEQQEHLHTE

Known epitopes from IEDB

PPERPFQATGITYTFPTDA	448	466	19
RMYLSSQAI	386	394	9

	Seq name	Peptide start	Peptide end	Peptide length	Peptide	N motif	C motif	Cleavage probability score	Cleavage probability percentile rank
1	TR|Q2PAY2|Q2PAY2_TOXGO RHOPTRY KINASE FAMILY P...	58	71	14	KPTSLDSQQHVADK	SKP	DKR	0.93322	0.00
2	TR|Q2PAY2|Q2PAY2_TOXGO RHOPTRY KINASE FAMILY P...	324	336	13	VPSDVVMLEGQPA	MVP	PAS	0.93166	0.02
3	TR|Q2PAY2|Q2PAY2_TOXGO RHOPTRY KINASE FAMILY P...	129	144	16	DGEVSGSAADSSSRPR	RDG	PRL	0.92236	0.03
4	TR|Q2PAY2|Q2PAY2_TOXGO RHOPTRY KINASE FAMILY P...	372	386	15	GDASVNKSEFGLVVR	FGD	VRM	0.90259	0.05
5	TR|Q2PAY2|Q2PAY2_TOXGO RHOPTRY KINASE FAMILY P...	447	459	13	EPPERPFQATGIT	YEP	ITY	0.89406	0.07

Partial Match

5	TR|Q2PAY2|Q2PAY2_TOXGO RHOPTRY KINASE FAMILY P...	447	459	13	EPPERPFQATGIT	YEP	ITY	0.89406	0.07

PPERPFQATGITYTFPTDA

>tr|A0A125YQL1|A0A125YQL1_TOXGM Rhoptry protein ROP18 OS=Toxoplasma gondii (strain ATCC 50611 / Me49) OX=508771 GN=ROP18 PE=4 SV=1

MFSVQRPPLTRTVVRMGLATLLPKTACLAVLNVALVFLLFQVQDGTGITLDPSKLDSKPT

SLDSQQHVADKRWPATVGHYKYLAGATESTRDVSLLEERAQHRVNAQETNQRRTIFQRLL

NLLRRRERDGEVSGSAADSSSRPRLSVRQRLAQLWRKAKSFFTRGIPRYFSQGRNRLRSL

RAQRRRSELFFEKADSGCVIGKRILAHMQEQIGQPQALGNSERLDRILTVAAWPPDVPER

FVSVTTGETRTLVRGAPLGSGGFATVYEATDVETNEELAVKVFMSEKEPTDETMRDLQRE

SFCYRNFSLAKTAKDAQERCRFMVPSDVVMLEGQPASTEVVIGLTTRWVPNYFLLMMRAE

TDMSKVISWVFGDASVNNSELGLVVRMYLSSQAIRLVANVQAQGIVHTDIKPANFLLLKD

GRLFLGDFGTYRINNSVGPAIGTPGYEPPERPFQTTDITYTFTTDAWQLGITLYCIWCKE

RPTPADGIWDYLHFADCPSTPELVQDLIRNLLNREPQKRMLPLQALETAAFNEMDSVVKR

AAQNFEQQEHLHTE

Known epitopes from IEDB

PPERPFQTTDITYTFTTDA	448	466	19

	Seq name	Peptide start	Peptide end	Peptide length	Peptide	N motif	C motif	Cleavage probability score	Cleavage probability percentile rank
1	TR|A0A125YQL1|A0A125YQL1_TOXGM RHOPTRY PROTEIN...	166	181	16	IPRYFSQGRNRLRSLR	GIP	LRA	1.33044	0.00
2	TR|A0A125YQL1|A0A125YQL1_TOXGM RHOPTRY PROTEIN...	51	66	16	DPSKLDSKPTSLDSQQ	LDP	QQH	0.97344	0.02
3	TR|A0A125YQL1|A0A125YQL1_TOXGM RHOPTRY PROTEIN...	58	71	14	KPTSLDSQQHVADK	SKP	DKR	0.93322	0.03
4	TR|A0A125YQL1|A0A125YQL1_TOXGM RHOPTRY PROTEIN...	324	336	13	VPSDVVMLEGQPA	MVP	PAS	0.93166	0.05
5	TR|A0A125YQL1|A0A125YQL1_TOXGM RHOPTRY PROTEIN...	129	144	16	DGEVSGSAADSSSRPR	RDG	PRL	0.92236	0.07

>tr|Q6RUA7|Q6RUA7_TOXGO Sporozoite-specific SAG protein OS=Toxoplasma gondii OX=5811 PE=1 SV=1

MSLLSRVAVLSVVALSFEMPMMLAADPEATSCETEGSSISFTVEKAGHVVRFNCPSTLEE

IKPAYEAGDSTKVCTTADCSNEAALKDVLKSASLAQAEGSGPSGGNDFTLTVDALPEAET

SVFFLCQRTGASRSARRLGTAVPSDKCGVHILVKAAPQAPVCSAQDHTLELQITAANSDT

SFVCGGTFNVIKPANAAKVLQGDSCETEVDLVSLVPHASRSALEQSGLIKLSVTDLPQQQ

QKLCYRCEDSSQKACKVLVTVSASHTSDAARLTAQAALGALLAVAGLVYMA

Known epitopes from IEDB

PFVCGGTFNV	182	190	10

	Seq name	Peptide start	Peptide end	Peptide length	Peptide	N motif	C motif	Cleavage probability score	Cleavage probability percentile rank
1	TR|Q6RUA7|Q6RUA7_TOXGO SPOROZOITE-SPECIFIC SAG...	215	230	16	VPHASRSALEQSGLIK	LVP	IKL	1.03380	0.00
2	TR|Q6RUA7|Q6RUA7_TOXGO SPOROZOITE-SPECIFIC SAG...	202	217	16	GDSCETEVDLVSLVPH	QGD	PHA	1.02224	0.03
3	TR|Q6RUA7|Q6RUA7_TOXGO SPOROZOITE-SPECIFIC SAG...	203	217	15	DSCETEVDLVSLVPH	GDS	PHA	0.98028	0.07

```
4       TR|Q6RUA7|Q6RUA7_TOXGO SPOROZOITE-SPECIFIC SAG...    142     158     17
        VPSDKCGVHILVKAAPQ       AVP     PQA     0.89211 0.10

5       TR|Q6RUA7|Q6RUA7_TOXGO SPOROZOITE-SPECIFIC SAG...    159     172     14
        APVCSAQDHTLELQ QAP      LQI     0.87779 0.13
```

```
>tr|Q9BJ38|Q9BJ38_TOXGO Surface antigen OS=Toxoplasma gondii OX=5811 GN=SAG3 PE=4 SV=1

MQLWRRRAAGPASLGRQSLPLGCFFAAFGLCVLSAILGTGEHGLFVAAGKSRSKITYFGT

LTQKAPNWYRCSSTRAKEEVVGHVTLNKEHPDMTIECVDDGLGGEFLPLEGATSSYPRVC

HIDAKDKGDCERNKGFLTDYIPGAKQYWYKIEKVENNGEQSVLYKFTVPWIFLPPAKQRY

KVGCRYPNHEYCFVEVTVEPTPPMVEGKRVTCGYPESGPVNLEVDLSKDANFIEIRCGEQ

HHPQPSTYTLQYCSGDSVDPQKCSPQSLTNIFYDYSSSWWKGKLNGPDGATLTIPPGGFP

EEDKSFLVGCSLTVDGPPFCNVKVRVAGNPRKWGRGGGGHPGSGGSQPETDGETQAGTES

SAGASSRMASVALAFLLGLLVHVAA
```

```
Known epitopes from IEDB

GLGGEFLPL                     101     109     9
```

```
        Seq name       Peptide start  Peptide end    Peptide length Peptide N motif C motif
        Cleavage probability score    Cleavage probability percentile rank

1       TR|Q9BJ38|Q9BJ38_TOXGO SURFACE ANTIGEN OS=TOXO...    316     331     16
        GPPFCNVKVRVAGNPR        DGP     PRK     1.32314 0.00

2       TR|Q9BJ38|Q9BJ38_TOXGO SURFACE ANTIGEN OS=TOXO...    286     301     16
        GPDGATLTIPPGGFPE        NGP     PEE     1.20386 0.02

3       TR|Q9BJ38|Q9BJ38_TOXGO SURFACE ANTIGEN OS=TOXO...    218     231     14
        GPVNLEVDLSKDAN SGP      ANF     1.04791 0.05

4       TR|Q9BJ38|Q9BJ38_TOXGO SURFACE ANTIGEN OS=TOXO...    286     300     15
        GPDGATLTIPPGGFP         NGP     FPE     1.03839 0.07

5       TR|Q9BJ38|Q9BJ38_TOXGO SURFACE ANTIGEN OS=TOXO...    329     342     14
        NPRKWGRGGGGHPG GNP      PGS     1.02111 0.10
```

Appendix C: Predicting cleavage sites

<u>Peptides downloaded from IEDB data:</u>

Selection criteria used …

Epitope: Linear peptides – Epitope source: Any – Host: Human – Assay: MHC Ligand (positive only) – MHC Restriction: Class II – Disease: Any

Output listed:

470,217	Epitopes
18,514	Antigens

1,197,102 Assays
802 References

Note: if 'Infectious' is used for the Disease setting, only 1997 epitopes are listed

Information extracted from the downloaded IEDB file

Number of peptides with antigen IDs: 442,830
Number of peptides with missing antigen IDs: 27,387
Number of peptides with duplicate antigen IDs: 424,805

Number of antigen IDs containing peptides: 18,025

Obtaining protein sequences for antigens containing peptides

UniProt sequences in a FASTA format obtained for 17,944 antigens (81 are obsolete)

Number of peptides not used: 27387 (missing antigen ID)
Number of different peptide lengths: 65
Number of species: 286 (97.11% of peptides have human parent proteins)

Total number of peptides with various lengths = 442,019 (associated with 17,944 proteins)

Creating training sequences for N-Terminal (15AA peptide length)

Number of ids: 17944

Number of input peptides	: 64673
Peptide position does not match start	: 5205
No start specified	: 1798
Peptide seq. contains invalid AA	: 8 (X and U)
Peptide not in parent sequence	: 3658
Parent sequence too short	: 1028

Number of valid peptides available : 52976

Creating training sequences for C-Terminal (15AA peptide length)

Number of ids: 17944

Number of input peptides	: 64673
Peptide position does not match end	: 5205
No end specified	: 1798
Peptide seq. contains invalid AA	: 3 (U)
Peptide not in parent sequence	: 3658

Parent sequence too short : 1800

Number of valid peptides available : 52209

Frequencies at position #2 at N-Terminal Flanking Region

AA: Observed: Expected: Ratio (Observed: Expected)

PFR2	P:5467:3311:1.651+++	A:4134:3766:1.098	G:4046:3485:1.161	E:3939:3782:1.041
	S:3832:4349:0.881	K:3660:3046:1.202+++	L:3536:5265:0.672---	
	D:3515:2601:1.351+++	V:3433:3247:1.057	R:2892:2918:0.991	T:2723:2876:0.947
	Q:2517:2511:1.002	N:2455:1960:1.252+++	I:2213:2352:0.941	F:1281:1912:0.67---
	H:1133:1313:0.862	Y:987:1414:0.698---	M:634:1123:0.565---	W:303:635:0.477---
	C:276:1101:0.25---			

Machine Learning Results

```
### N-terminal ###

Error matrix for the Random Forest model on training_data.txt [validate] (counts):

      Predicted
Actual    0    1
     0 4483 3388
     1 3290 4731

Error matrix for the Random Forest model on training_data.txt [validate]
(proportions):

      Predicted
Actual    0    1 Error
     0 0.28 0.21  0.43
     1 0.21 0.30  0.41

Overall error: 42%, Averaged class error: 42%

58% accuracy


Variable Importance
===================

         0     1 MeanDecreaseAccuracy MeanDecreaseGini
PFR2 23.16 44.32                43.88          1684.43
PFR1 17.56 45.41                42.41          1610.55
P10  19.65 40.71                41.61          1738.22
PFR3  3.93 24.63                20.34          1772.54
P8    0.79 19.98                15.72          1862.57
P1    2.08 19.22                15.11          1816.69
P9   -0.02 21.21                14.81          1820.44
P7   -0.67 20.14                14.24          1811.58
P6    0.16 19.41                13.79          1884.02
P3    0.92 18.89                13.78          1837.07
P5    1.97 17.41                13.77          1836.12
P4    0.84 17.56                12.75          1866.33
P2    0.09 18.00                12.53          1883.63


Error matrix for the SVM model on training_data.txt [validate] (counts):
```

```
      Predicted
Actual    0    1
     0 3990 3938
     1 3727 4246

Error matrix for the SVM model on training_data.txt [validate] (proportions):

      Predicted
Actual    0    1 Error
     0 0.25 0.25  0.50
     1 0.23 0.27  0.47

Overall error: 48%, Averaged class error: 48%

Error matrix for the Neural Net model on training_data.txt [validate] (counts):

      Predicted
Actual    0    1 <NA>
  0    4123 3805    0
  1    3919 4054    0
  <NA>    0    0   22

Error matrix for the Neural Net model on training_data.txt [validate]
(proportions):

      Predicted
Actual    0    1 Error
     0 0.26 0.24  0.48
     1 0.25 0.25  0.49

Overall error: 49%, Averaged class error: 48%

### C-terminal ###

Error matrix for the Random Forest model on L15_training [validate] (counts):

      Predicted
Actual    0    1
     0 4432 3471
     1 3236 4523

Error matrix for the Random Forest model on L15_training [validate] (proportions):

      Predicted
Actual    0    1 Error
     0 0.28 0.22  0.44
     1 0.21 0.29  0.42

Overall error: 43%, Averaged class error: 43%

57.2% accuracy



Variable Importance
===================

         0     1 MeanDecreaseAccuracy MeanDecreaseGini
PFR3 19.48 45.23                44.32          1623.83
PFR2  8.45 31.84                27.84          1574.58
P1    9.36 28.47                27.73          1648.96
PFR1  4.54 21.81                18.20          1672.36
P7    0.73 18.38                13.31          1836.30
P4   -0.80 18.12                13.08          1841.82
P2   -2.20 19.38                12.72          1832.81
P6   -0.51 16.59                11.70          1820.54
P3    0.27 15.48                11.28          1851.68
P8    0.74 15.45                11.18          1818.28
```

```
P10   0.77 14.91                11.11          1828.82
P5   -0.45 15.02                10.09          1875.26
P9   -0.71 13.73                 9.56          1858.57
```

```
Error matrix for the Random Forest model on L15_training [validate] (counts):

      Predicted
Actual    0    1
     0 8813 6878
     1 6474 9160

Error matrix for the Random Forest model on L15_training [validate] (proportions):

      Predicted
Actual    0    1 Error
     0 0.28 0.22  0.44
     1 0.21 0.29  0.41

Overall error: 43%, Averaged class error: 42%
```
